# Cell wall-associated ROOT HAIR SPECIFIC 10, a proline-rich receptor-like kinase, is a negative modulator of Arabidopsis root hair growth

**DOI:** 10.1093/jxb/erw031

**Published:** 2016-02-16

**Authors:** Youra Hwang, Hyodong Lee, Young-Sook Lee, Hyung-Taeg Cho

**Affiliations:** Department of Biological Sciences, Seoul National University, Seoul 151-742, Korea

**Keywords:** Arabidopsis, cell elongation, cell wall, receptor-like kinase, root hair, tip growth.

## Abstract

Plant cell growth is restricted by the cell wall, and cell wall dynamics act as signals for the cytoplasmic and nuclear events of cell growth. Among various receptor kinases, ROOT HAIR SPECIFIC 10 (RHS10) belongs to a poorly known receptor kinase subfamily with a proline-rich extracellular domain. Here, we report that RHS10 defines the root hair length of *Arabidopsis thaliana* by negatively regulating hair growth. RHS10 modulates the duration of root hair growth rather than the growth rate. As poplar and rice RHS10 orthologs also showed a root hair-inhibitory function, this receptor kinase-mediated function appears to be conserved in angiosperms. RHS10 showed a strong association with the cell wall, most probably through its extracellular proline-rich domain (ECD). Deletion analysis of the ECD demonstrated that a minimal extracellular part, which includes a few proline residues, is required for RHS10-mediated root hair inhibition. RHS10 suppressed the accumulation of reactive oxygen species (ROS) in the root, which are necessary for root hair growth. A yeast two-hybrid screening identified an RNase (RNS2) as a putative downstream target of RHS10. Accordingly, *RHS10* overexpression decreased and *RHS10* loss increased RNA levels in the hair-growing root region. Our results suggest that RHS10 mediates cell wall-associated signals to maintain proper root hair length, at least in part by regulating RNA catabolism and ROS accumulation.

## Highlight

RHS10 mediates cell wall-associated signals to maintain proper root hair length, potentially by regulating RNA catabolism and ROS accumulation.

## Introduction

A root hair develops in a stepwise manner through cell fate determination, initiation, bulge formation, and tip growth (Grierson and Schiefelbein, 2002, 2008). Three different distribution patterns of hair/non-hair cells (i.e. random, longitudinal, and radial patterning), which derive from different fate-determining mechanisms, have been observed in vascular plants (Dolan, 1996; Clowes, 2000; Schiefelbein, 2000). Although this upstream fate determination step has evolutionarily diverged, the downstream hair-forming morphogenetic steps are likely to be conserved throughout the vascular plant lineage (Kim *et al.*, 2006). ROOT HAIR DEFECTIVE 6 [RHD6; a basic helix–loop–helix (bHLH) transcription factor] has been recognized as a prime positive modulator of root hair initiation in root hair cells of Arabidopsis (Masucci and Schiefelbein, 1996; Menand *et al.*, 2007), and its molecular function is conserved even between moss and Arabidopsis (Menand *et al.*, 2007). RHD6 directly regulates two downstream bHLH transcription factors, RHD6-LIKE2 (RSL2) and RHD6-LIKE 4 (RSL4), and RSL4 regulates various morphogenetic genes, including the genes involved in root hair morphogenesis (Yi *et al.*, 2010).

In a previous study, we identified a *cis*-element (RHE for Root Hair-specific *cis*-Element) that directs root hair cell-specific gene expression, and demonstrated that RHE is structurally and functionally conserved in angiosperms (Kim *et al.*, 2006). In an effort to identify more root hair-specific genes in the Arabidopsis genome, which include an RHE in their promoter region, we acquired 19 root hair-specific (*RHS*) genes that were confirmed by an *in planta* promoter:reporter assay (Won *et al.*, 2009). *RHS* genes include morphogenetic genes such as those for cell wall dynamics, kinases, and signaling-related genes, and their loss of function or overexpression altered root hair elongation and polarity (Won *et al.*, 2009). Among these *RHS* genes, *RHS10*, which encodes a proline-rich extensin-like receptor kinase (PERK), had a negative role in root hair elongation or tip growth (Won *et al.*, 2009).

Receptor-like kinases (RLKs) that localize to the plasma membrane (PM) are thought to mediate extracellular signals to the cytoplasm and nucleus. The Arabidopsis genome includes >600 members of RLKs that are classified into 46 subfamilies (Shiu and Bleekcker, 2003). Although some subfamilies belonging to leucine-rich repeat (LRR) RLKs have been relatively well characterized, the molecular and biological functions of most RLKs remain to be studied. At least four RLK subfamilies have been implicated in sensing cell wall integrity: wall-associated kinases (WAKs), lectin receptor kinases (LecRKs), THESEUS1 (THE1), and PERKs (Humphrey *et al.*, 2007).

The PERK family kinase was first identified in *Brassica napus* (*BnPERK1*; Silva and Goring, 2002), and the Arabidopsis genome includes 15 PERK homologs (Nakhamchik *et al.*, 2004). All of the 15 PERK members of Arabidopsis share a common domain structure: a proline-rich extensin-like extracellular domain (ECD), a transmembrane domain (TM), and a serine/threonine kinase domain in order from the N-terminus (Nakhamchik *et al.*, 2004). The extensin-like domain of a PERK includes several repeats of SP_3–5_, but, unlike typical extensin, lacks an adjacent YXY (X is any amino acid residue) motif for cross-linking (Showalter *et al.*, 2010). The existence of an extensin-like domain led to the idea that PERKs could be associated with the cell wall (Bai *et al.*, 2009a). Two PERKs, BnPERK1 (Silva and Goring, 2002) and Arabidopsis PERK4 (Bai *et al.*, 2009a), were shown to localize to the PM and have serine/threonine kinase activity.

A few studies have characterized the biological function of PERKs. Antisense-mediated suppression of PERK1-related genes caused diverse phenotypic effects, such as loss of apical dominance, increased branching, and floral organ defects, and overexpression of *BnPERK1* increased the life span, lateral shoots, and seed set in Arabidopsis (Haffani *et al.*, 2006). Inhibition of Arabidopsis root cell elongation was observed in *perk4*, *perk13*, and triple *perk8/9/10* mutants (Humphrey *et al.*, 2007; Bai *et al.*, 2009a). Recent studies by Bai *et al.* (2009a, b) showed that Arabidopsis PERK4 is required for abscisic acid (ABA)-mediated gene regulation, Ca^2+^ channel opening, and inhibition of root growth and seed germination. Another study by Humphrey *et al.* (2015) demonstrated that the kinase domain of PERKs interacts with AGC family protein kinases. Our previous study demonstrated that loss of function of RHS10/PERK13 enhanced and its overexpression inhibited root hair growth (Won *et al.*, 2009). Arabidopsis *IGI1*/*PERK12* has been implicated in branching and growth of the shoot (Hwang *et al.*, 2010).

Although a few of the aforementioned studies have demonstrated the basic aspects of PERK function, further questions remain to be answered; for example, how the ECD works, what the PERK downstream process is, how PERKs interact with other signaling pathways, and whether cell wall-associated PERK function is evolutionarily conserved. In this study, we characterized these molecular and biological functions of RHS10/PERK13 during root hair growth. Our data demonstrate that minimal proline residues in the ECD are sufficient for RHS10 function, RHS10 affects root hair growth by regulating reactive oxygen species (ROS) levels and RNA metabolism, and RHS10 function during root hair growth has been conserved in angiosperms.

## Materials and methods

### Plant material and growth conditions

*Arabidopsis thaliana* wild type (WT, Columbia) was used for transformation of transgene constructs unless stated otherwise. Arabidopsis plants were transformed using *Agrobacterium tumefaciens* strain C58C1 (pMP90). Transformed plants were selected on hygromycin-containing plates (50 µg ml^–1^). All seeds were grown on agarose plates containing 4.3g ml^–1^ Murashige and Skoog (MS) nutrient mix (Duchefa), 1% sucrose, 0.5g ml^–1^ MES at pH 5.7 with KOH, and 0.8% agarose. Seeds were cold treated before germination at 23 °C under a 16h/8h light/dark photoperiod. For observation of root hairs, homozygous transformants were planted on antibiotic-free media, and T_1_ and T_2_ lines were planted on hygromycin-containing media. Hygromycin did not significantly interfere with root hair development, as shown with the control *ProE7:YFP* transformants in each experiment. Two control lines were adopted: WT for mutant analysis and *ProE7:YFP* for transgenic analyses on the medium including hygromycin. Unless specifically mentioned, T_2_ or homozygous transformants were used.

### Observation of root hair phenotypes and measurement of root hair length

Root hair phenotypes were observed under a stereomicroscope (MZ FLIII, Leica, Heerbrugg, Switzerland). Root hair length was measured as described in Lee and Cho (2006) with modifications. The root was digitally photographed using a stereomicroscope at ×40–50 magnification. The hair length of 8–10 consecutive hairs protruding perpendicularly from each side of the root, for a total of 16–20 hairs from both sides of the root, was calculated using LAS software V2.8.1 (Leica). Root hairs were observed with 3-day-old seedlings.

### Construction of transgenes

The binary vector *pCAMBIA1300-NOS* with modified cloning sites (Lee *et al.*, 2010) was used for transgene construction. The At*EXPA7* promoter (*ProE7*; Cho and Cosgrove, 2002; Kim *et al.*, 2006) was used for root hair-specific expression. *ProE7:YFP* and *ProE7:PIN3:GFP* (Lee and Cho, 2006; Ganguly *et al.*, 2010) and *ProE7:RHS10ox* and *ProE7:axr2-1* (Won *et al.*, 2009) were described previously.

For the *ProE7:RHS10:GFP* construct, a genomic fragment of *RHS10* was obtained by PCR using the primer sets listed in Supplementary Table S1 at *JXB* online and Arabidopsis genomic DNA as a template. The PCR product was cloned into *Sal*I/*Nco*I sites upstream of the *GFP* (green fluorescent protein) gene, producing a *RHS10:GFP* fusion. For *ProE7:PERK8*, *ProE7:RNS2*, *ProE7:OsRHS10* (Os03g37120 and Os06g29080), and *ProE7:PtRHS10* constructs, the genomic fragments were obtained by PCR using the primer sets listed in Supplementary Table S1 and *A. thaliana*, *Oryza sativa*, and *Populus trichocarpa* genomic DNA as templates. For *ProE7:PERK8* and *ProE7:RNS2*, the PCR products were cloned into *Sal*I/*Bam*HI sites, and Os*RHS10* (Os03g37120 and Os06g29080) and Pt*RHS10* PCR products were inserted into *Bgl*II/*Nco*I and *Sal*I/*Kpn*I sites, respectively, downstream of *ProE7*.

For the deletion analysis of the RHS10 ECD, the serial deletion fragments (D1–D5) were generated by PCR using the primers listed in Supplementary Table S1 and inserted into *Bgl*II/*Nco*I sites of the *pCAMBIA1300* vector containing *ProE7*. The nucleotides (RHS10 Sl-MF/RHS10 Bg2-MR in Supplementary Table S1) corresponding to the first four amino acid residues, including the start codon, were inserted into the *Sal*I/*Bgl*II sites before the deletion inserts.

For LRX2-Ri and ROL1-Ri-1/2 construction, the RNAi target regions of *LRX2* and *ROL1* cDNA were amplified using PCR with the primer sets listed in Supplementary Table S1. Each RNAi target region was inserted into *Xho*I/*Eco*RI and *Hin*dIII/*Xba*I sites of the *pHannibal* vector to generate a sense and antisense construct, respectively. For the final RNAi construction in a binary vector, the *Cauliflower mosaic virus* 35S promoter (*Pro35S*) was inserted into the *Hin*dIII/*Sal*I site of the *pCAMBIA1300* vector, and the *Xho*I/*Xba*I fragments of the RNAi inserts from the *pHannibal* vector were transferred into the *Sal*I/*Xba*I sites downstream of *Pro35S*.

In order to express RNS2 and RHS10 proteins in *Escherichia coli* for protein blot analysis and *in vitro* kinase assay, the cDNA sequences of *RNS2* and *RHS10* kinase domain were amplified by PCR from the Arabidopsis seedling cDNA library using the primer sets listed in Supplementary Table S1. The PCR products were cloned into *Eco*RI/*Sal*I restriction sites of the *pGEX-4T-1* vector (GE Healthcare, Inchon, Korea), which generated fusion proteins with glutathione *S*-transferase (GST) at the N-termini of the RNS2 and RHS10 kinase domain.

All constructs were confirmed by nucleotide sequencing and introduced into Arabidopsis plants by the *Agrobacterium*-mediated floral dipping method. Transgene insertion in the Arabidopsis transformants was confirmed by PCR analysis using transgene-specific primers.

### Microscopic observation of fluorescent proteins

Fluorescence from GFP (green) and FM4-64 (red) was observed using an LSM 510 confocal laser scanning microscope (Carl Zeiss). Localization of the RHS10:GFP and PIN3:GFP fusion proteins was observed in 4-day-old seedlings. To observe brefeldin A (BFA) compartment formation, the transgenic seedlings were co-treated with cycloheximide (50 μM) and BFA (25 μM) for 1–2h before observation. The PM and endocytosis tracer FM4-64 (2 μM) was applied for <3min for PM marking and ~15min for BFA body marking. The control liquid medium included the same amount of DMSO, which was used to dissolve BFA. To estimate the cell wall/PM ratio of RHS10:GFP and PIN3:GFP, seedling roots were plasmolyzed with 0.45M mannitol for 5min before observation. The fluorescence signal intensities from the cell wall and PM were estimated from the same area of a ROI (region of interest) of the cell wall or the PM region after taking confocal micropictographs. The calculation of fluorescence signal intensities was performed using the Adobe Photoshop histogram menu as described previously (Kim *et al.*, 2006; Won *et al.*, 2010).

### Preparation of membrane proteins and protein blot analysis

Total cytosolic and membrane proteins were isolated from transgenic Arabidopsis seedlings (*ProE7:GFP* and *ProE7:RHS10:GFP*). Four-day-old seedlings were ground in ice-cold homogenization/extraction buffer [250mM HEPES-KOH (pH7.0), 0.5M NaCl, 0.1M EDTA, 50mM potassium acetate, phenylmethylsulfonyl fluoride, and protease inhibitors]. The resultant total cellular proteins (the supernatant of low speed centrifugation) were subjected to centrifugation at 10 000 *g* for 1h to obtain microsomal pellets and ‘cytosolic proteins’ in the supernatant. Pellets were re-suspended in microsome buffer with 1% Triton X-100 and separated into supernatant and pellet fractions at 10 000 *g*. This supernatant fraction is denoted as ‘microsome proteins’. GFP (from *ProE7:GFP*) and RHS10:GFP (from *ProE7:RHS10:GFP*) from cytosolic (C) and microsomal (M) parts were analyzed by western blot analysis using anti-GFP antibody. An equal amount of each protein sample was separated by SDS–PAGE, transferred into a nitrocellulose membrane (Amersham Biosciences, Corston, Bath, UK), and probed with 1/500-diluted anti-GFP rabbit IgG horseradish peroxidase (HRP)-conjugated antibody (Invitrogen, Seoul, Korea). Chemiluminescence detection was performed with Pierce ECL western blotting substrate (Thermo Scientific Inc., Waltham, MA, USA) on a chemiluminescence imaging system (Davinch-Chem, Corebio, Seoul, Korea).

### Generation of multiple mutants

Multiple mutants and transformants used to show the genetic relationship with RHS10 were generated by crossing individual lines, and their homozygosity was verified by molecular and physiological genotyping.

### Measurement of ROS

A ROS-sensitive fluorescent dye [2′,7′-dichlorodihydrofluorescein diacetate (H_2_DCFDA), Invitrogen] was used to estimate ROS levels in the root. Three-day-old seedlings were incubated with 3 μM H_2_DCF-DA (dissolved in DMSO, 0.0045% final concentration) for 60min at 4 °C, washed with 0.1mM KCl and 0.1mM CaCl_2_ (pH 6.0), and incubated for 60min at 22 °C before observation. The green fluorescence images were taken by a fluorescence stereomicroscope (MZ FLIII, Leica), and the ROS levels were estimated by quantifying green fluorescence in the root using the histogram function of Adobe Photoshop CS6 (Adobe Systems) as described previously (Cho and Cosgrove, 2002; Kim *et al.*, 2006). The same area of the ROI was used for different root samples. The experiment was repeated twice, and 11–23 seedlings were observed for each genotype.

### Yeast two-hybrid screening and assay

A yeast two-hybrid (Y2H) screening and assay was performed using the Matchmaker Yeast Two-Hybrid System (Clontech, USA). The RHS10 kinase domain (amino acids 256–710) was PCR-amplified using the primer sets in Supplementary Table S1 and cloned into the *Nco*I/*Pst*I sites of the bait *pGBKT7* vector, and the Arabidopsis seedling cDNA library was cloned into the *pGADT7* library vector. Y2H screening processes were conducted according to the manufacturer’s manual. About 79 putative targets of RHS10 kinase were screened from Quadruple Dropout (QDO)/X-α-gal selection medium. Isolated positive plasmids were subjected to nucleotide sequence analyses to confirm gene identities. Root hair-related genes were further screened using cell type-specific expression databases such as Arabidopsis eFP Browser (BAR, http://bar.utoronto.ca/welcome.htm) and Genevestigator (https://www.genevestigator.com). The interaction between the RHS10 kinase domain and several final interactor candidates was re-confirmed with a targeted Y2H assay. The RHS10 N-terminal bait (amino acids 1–236.) was PCR amplified using the primer sets in Supplementary Table S1 and cloned into the *Nco*I/*Pst*I sites of the bait *pGBKT7* vector. In order to suppress background HIS3 activities, 0–70mM 3-amino-1,2,4-triazole (3-AT) was used.

### Quantification of cellular RNA contents

Whole seedlings were stained for 3h with 5 μM SYTO RNASelect green (Invitrogen) in liquid MS medium at room temperature (Hillwig *et al.*, 2011), washed three times with fresh MS medium, and observed under a stereo- or confocal microscope used to produce green fluorescent root images as described above. Quantification of root RNA content, which was represented by green fluorescence, was performed using the Adobe Photoshop histogram menu with the root fluorescence images as described previously (Kim *et al.*, 2006; Won *et al.*, 2010).

### Accession numbers

Sequence data or mutants from this article can be found in the Arabidopsis Information Resource (https://www.arabidopsis.org/) database under the following accession numbers: AT1G70460 (*RHS10*), AT3G24550 (*PERK1*), AT4G34440 (*PERK5*), AT5G38560 (*PERK8*), AT1G26150 (*PERK10*), PtEEE90055 (Pt *RHS10*), AT2G39780 (*RNS2*), AT1G66470 (*RHD6*), AT5G51060 (*RHD2*), AT1G70940 (*PIN3*), AT1G12040(*LRX1*), AT1G62440 (*LRX2*), AT3G23050 (*AXR2*), AT5G03280 (*EIN2*), AT4G34580 (*COW1*), AT1G78570 (*ROL1*), *rhs10* (SALK_075892), *rns2* (SALK_069588), *perk1* (CS827714), *perk5* (CS842269), *perk8* (CS311575), and *perk10* (SALK_090553).

### Supplementary methods

The methods for supplementary data are described in the ‘Supplementary methods’.

## Results

### Loss of RHS10 extends the tip-growing period of root hairs

**Fig. 1.**
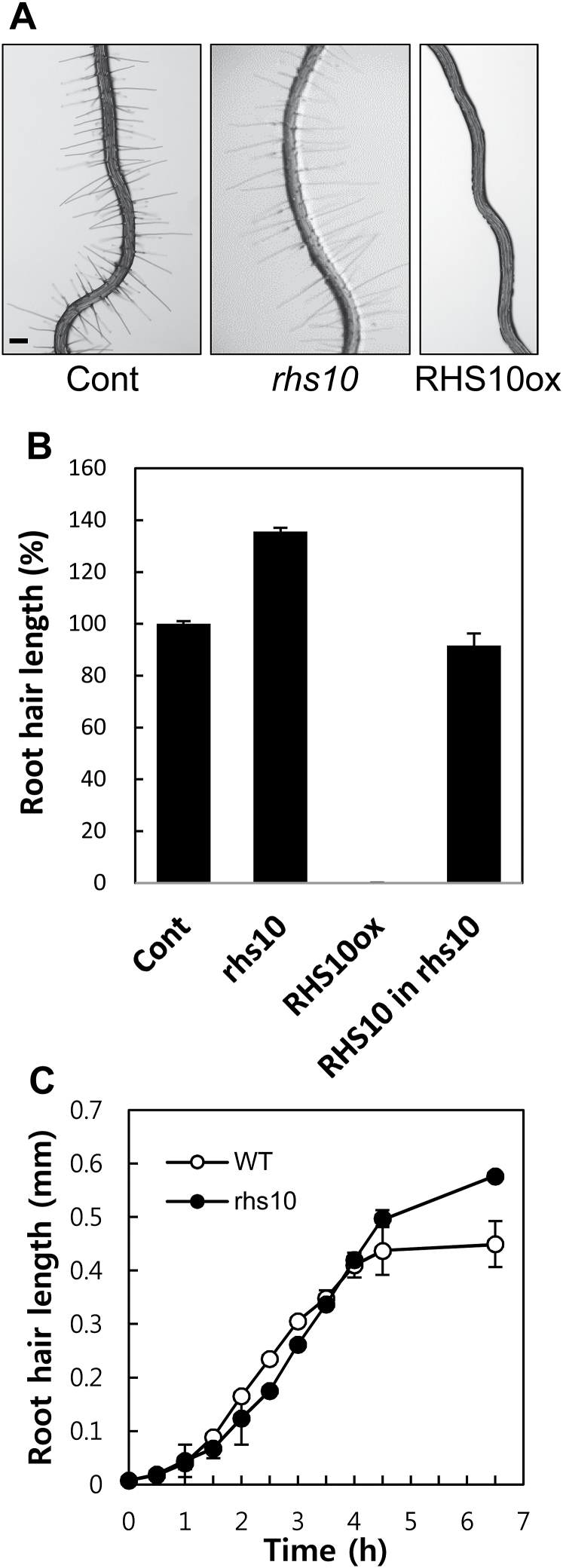
The inhibitory function of RHS10 on root hair growth. (A) Representative root images showing the role of RHS10 on root hair growth. While the loss-of-function *rhs10* mutant grows longer hairs, root hair-specific *RHS10* overexpression lines (RHS10ox, *ProE7:RHS10*) grow short, or no hairs. The scale bar is 100 μm in all images. Cont, control (*ProE7:YFP*). (B) Root hair lengths of Cont, *rhs10*, RHS10ox, and complemented [*ProRHS10:RHS10* (RHS10) in *rhs10*] lines. Data represent means ±SE from 641 (40 roots, Cont), 624 (39 seedlings, *rhs10*), 371 (22 seedlings, RHS10ox), and 2696 (168 seedlings from 10 independent lines of RHS10 in *rhs10*) root hairs. (C) Growth dynamics of single root hairs over time. Data represent the mean ±SE for 5–6 individual root hairs.

The loss-of-function *rhs10* mutant (SALK_075892) seedling grew considerably longer root hairs (50–60%) than the control seedling, and root hair-specific overexpression of *RHS10* under the *EXPANSIN A7* promoter (*ProE7*; Cho and Cosgrove, 2002) greatly inhibited root hair growth (Fig. 1A, B; Won *et al.*, 2009). Three other independent insertion mutant lines of *RHS10* also showed consistently longer root hair phenotypes (Supplementary Fig. S1). A complementation of *RHS10* under its own promoter in the mutant almost restored WT-level root hair growth (Fig. 1B). Whereas many kinase-related *RHS* genes are implicated in root hair morphogenetic processes such as hair branching and waving, *RHS10* influences only hair tip growth. The longer root hair phenotype of *rhs10* seems to be due to prolonged tip growth. Up to 4h after the initiation of tip growth from a minute bulge, both the WT and the *rhs10* mutant grew the hair at a similar speed (Fig. 1C); however, after this occurred and while the WT hair almost stopped growing, the *rhs10* hair still maintained a specific growth rate (Fig. 1C), suggesting that the *rhs10* mutant grows longer hairs by lengthening the duration of tip growth but not the growth rate.

### RHS10 localizes to the PM and exhibits association with the cell wall in root hair cells

**Fig. 2.**
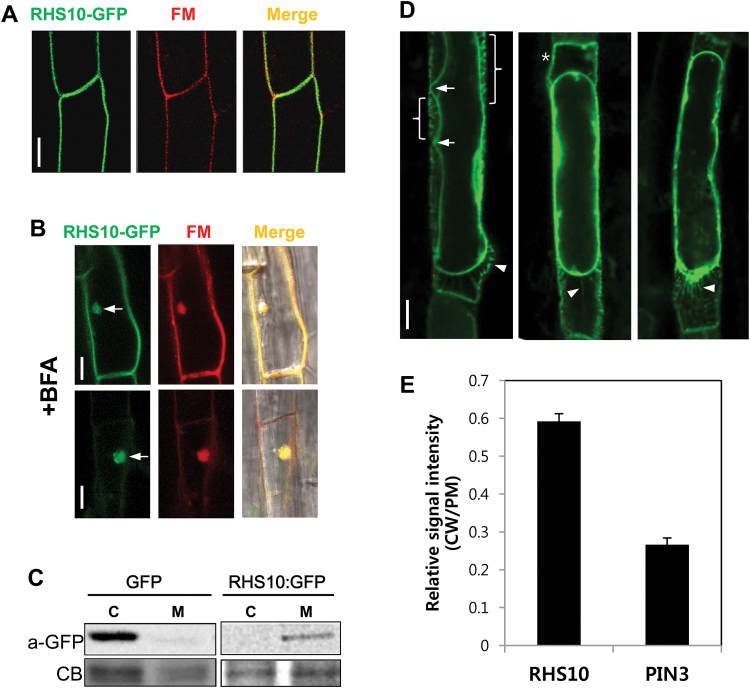
The analyses of the subcellular localization of RHS10. (A) The RHS10 (*ProE7:RHS10:GFP* in *rhs10*) green signal overlaps with the PM-marked FM4-64 red signal (FM). The transformant seedling was treated with FM4-64 for <3min. The scale bar is 10 μm in all images. (B) RHS10 forms BFA compartments indicating its PM origin. Seedlings were incubated with BFA for 1h (upper panels) or 2h (lower panels) in the presence of cycloheximide. Green RHS10 signals overlap with the endocytosed red FM4-64 signals (FM) in the BFA compartment. Arrows indicate the BFA compartment including RHS10:GFP. The scale bar is 10 μm in all images. (C) RHS10 is abundant in the membrane fraction. The protein blot analysis with the anti-GFP antibody shows that the RHS10:GFP fusion protein is mostly found in the total membrane fraction (M), whereas soluble GFP was found in the cytosolic fraction (C). (D) Three independent trichoblast cells showing RHS10:GFP signals, which are associated with the cell wall during plasmolysis. RHS10 remains in (bracket and asterisk), or forms Hechtian strands (arrow heads) with, the cell wall after plasmolysis using 0.45M mannitol. The scale bar is 10 μm in all images. (E) RHS10 tends to localize more frequently in the cell wall than PIN3. Relative signal intensities of RHS10:GFP (*ProE7:RHS10:GFP*) and PIN3:GFP (*ProE7:PIN3:GFP*) were observed in the cell wall and PM after plasmolysis, and the intensity ratio between the cell wall and PM (CW/PM) was calculated. Data represent the mean ±SE from 98 ROIs (from 50 cells of 10 seedlings) for each.

To determine the subcellular localization of RHS10, the RHS10:GFP fusion protein was expressed under *ProE7* and analyzed in the root hair cell. Root hairs specifically expressed the RHS10:GFP fusion protein, which could inhibit root hair growth, but with a somewhat lower efficiency than that of native RHS10, which could be due to partial functional hindrance of the kinase domain where GFP fused (Supplementary Fig. S2). By using confocal microscopy, the RHS10:GFP signal was observed along the root hair cell boundary, and it overlapped with the FM4-64 dye signal (Fig. 2A). The endocytotic tracer FM4-64 stains the PM when applied for a short duration (i.e. <3min), indicating that RHS10 localizes to the PM.

Internal accumulation of a protein in the BFA-induced compartment is cytological evidence supporting PM localization of the protein (Ganguly *et al.*, 2012). Similarly, to what has been shown for PIN-FORMED proteins (Geldner *et al.*, 2001), RHS10 obviously accumulated into the FM4-64-overlapping BFA compartments, and the RHS10 signal in the PM greatly decreased following prolonged BFA treatment (Fig. 2B), indicating that RHS10 is localized in the PM, and recycles between the PM and endosomes.

The protein blot analysis with proteins from the membrane and cytosolic fractions further supports PM localization of RHS10. Whereas GFP was detected mostly in the cytosolic fraction, RHS10:GFP was detected predominantly in the membrane fraction (Fig. 2C).

Because RHS10 includes extensin-like motifs in its putative ECD, we examined whether RHS10 shows a cell wall association, using the plasmolysis method. When the root hair cell of the RHS10:GFP-expressing transformant seedling was plasmolyzed by 0.45M mannitol, RHS10:GFP signals were observed not only in the PM, but also in the cell wall (Fig. 2D). Furthermore, even after plasmolysis, the RHS10:GFP signal exhibited connections (i.e. Hechtian strands) between the PM and cell wall (Fig. 2D). Many speckle-like RHS10:GFP signals were also observed in the cell wall, which potentially represent RHS10:GFP proteins left behind in the cell wall after plasmolysis and/or the strong RHS10–cell wall adhesion spots. PIN3 showed no detectable polarity in the root hair cell, and PIN3 consistently exhibited a very weak cell wall association (Supplementary Fig. S3). When quantified, the degree of cell wall association (i.e. the relative signal ratio of the protein in the cell wall over the PM protein) was considerably higher for RHS10 than for PIN3 in the root hair cell (Fig. 2E). This observation provides further evidence for the strong association of RHS10 with the cell wall, most probably through its proline-rich ECD.

### Minimal proline residues of the ECD are sufficient for RHS10 function in root hair inhibition

**Fig. 3.**
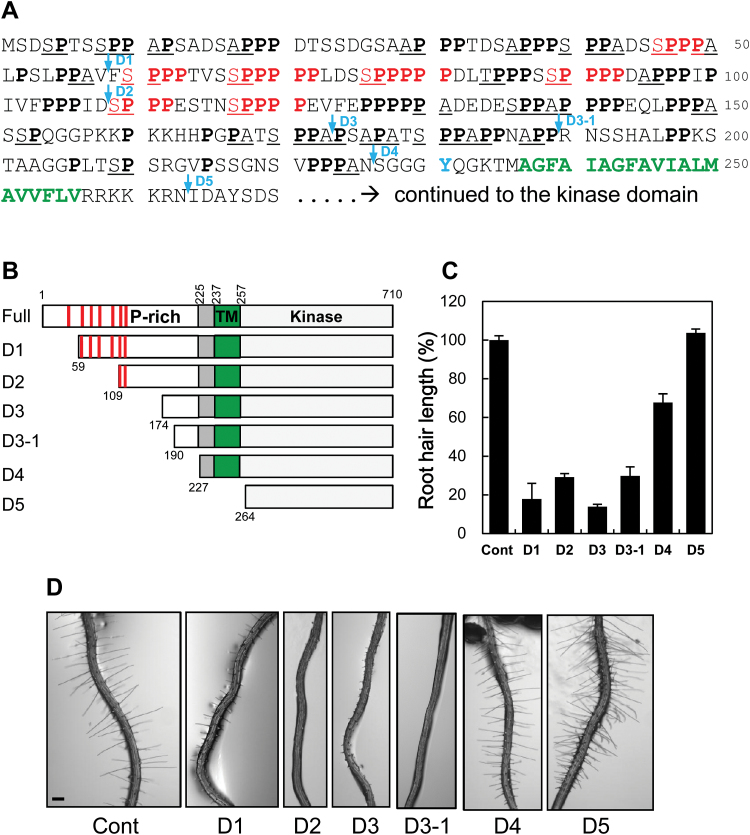
Deletion analysis of the extracellular domain of RHS10 during root hair growth. (A) The amino acid sequence of the N-terminal region (proline-rich extracellular domain and transmembrane domain) of Arabidopsis RHS10. Proline residues are in bold, extensin-like SPx repeats are in red, arabinogalactan protein (AGP) repeats (AP/PA/SP/TP) are underlined, and the transmembrane domain is in green and bold. The deletion positions for the N-terminal deletion analysis (D1–D5 as shown in B) are marked by arrows. The transmembrane domain was predicted using TMHMM Server v. 2.0 (http://www.cbs.dtu.dk/services/TMHMM-2.0/). (B) N-terminal deletion constructs of RHS10. Red bars, extensin motifs; P-rich, proline-rich region; TM, transmembrane-spanning domain. (C) The root hair-inhibitory effect by RHS10 deletion constructs. Root hair lengths were observed from T_1_ lines of root hairs specifically expressing RHS10 deletion constructs (*ProE7:ΔRHS10*). Cont, control (*ProE7:YFP*). Data represent the mean ±SE from 93–1228 root hairs (364 root hairs per construct on average) from 5–75 seedlings (22 seedlings per construct on average). (D) Representative root images of RHS10 deletion transformants. The scale bar is 100 μm in all images.

Due to the proline-rich extensin-like domain, PERKs have been suggested to be associated with the cell wall. The extensin-like putative ECD (composed of 236 residues before the TM domain) of RHS10 includes 88 proline residues (37.3% of the 236 residues) and seven extensin motifs (SP_3–5_), where the proline-rich region stretches up to the 224th residue from the N-terminus (Fig. 3A). In order to determine which region of the ECD is necessary for RHS10 function in terms of root hair inhibition, we generated serial N-terminal deletion constructs (D1–D5) of RHS10 (Fig. 3A, B), expressed these truncated forms in the root hair cell using *ProE7*, and estimated root hair length of independent transformant lines to reflect RHS10 activity.

In the first round deletion, considerable RHS10-mediated root hair inhibition [~14% of the control (*ProE7:YFP*) level] was observed up to the D3 (amino acids 173) deletion, which excluded all seven extensin-like motifs and 88% of proline residues (Fig. 3C, D). On the other hand, the D4 (amino acids 226) deletion carrying no proline residue greatly restored root hair growth (68% that of the control). The transformant only expressing the soluble kinase domain (deletion D5) grew root hairs the same size as the control. Because the region between D3 and D4 turned out to be critical for root hair inhibition activity by RHS10, we made one more deletion in this region (D3-1) that excludes 16 more residues from D3; however, the D3-1 deletion also showed the same intensity of root hair inhibition as D2 (Fig. 3C, D). D3-1 still includes eight proline residues (Fig. 3A). This deletion analysis of the ECD suggests that the extensin-like (SP_3–5_) repeats are not essential, but a few proline residues or other motifs probably could be critical for the activity of RHS10. The D4 deletion of RHS10, which includes no proline residue in its ECD, still had ~30% root hair inhibition activity (Fig. 3C). This level of activity might reflect basal RHS10 kinase activity without proline-involved extracellular signaling. Almost no root hair inhibition by the deletion of D5 (only containing the kinase domain) indicates that kinase activity on the downstream effectors requires ECD-mediated signaling from the cell wall.

### RHS10 function is likely to be conserved in angiosperms

Although cell wall compositions vary by species (Carpita and Gibeaut, 1993), and thus the cell wall-associated events could be different among species, root hair-specific gene regulation and root hair morphogenetic processes seem to be conserved in the angiosperm lineage (Kim *et al.*, 2006). In this context, we wondered whether there are RHS10-homologous proteins in other angiosperms and if they function like Arabidopsis RHS10 in the root hair cell. Therefore, we screened RHS10-homologous protein sequences from a monocotyledonous grass family and other eudicot members, and analyzed the phylogenetic relationship to identify RHS10-orthologous sequences (Supplementary Fig. S4). In this study, we focused on rice and poplar orthologs. The AtRHS10 clade included two Arabidopsis paralogs, and two rice and two poplar orthologous sequences (Supplementary Fig. S4). Here, we use the terms orthologs and paralogs following the definition of Koonin (2005). We chose two rice and one poplar RHS10-orthologous sequences; Os03g37120 and Os06g29080 for rice and PtEEE90055 (PtRHS10 hereafter) for poplar. These RHS10-orthologous sequences possessed all three proline-rich (with multiple extensin motifs), TM, and kinase domains, which are similar to AtRHS10 (Supplementary Figs S5, S6).

**Fig. 4.**
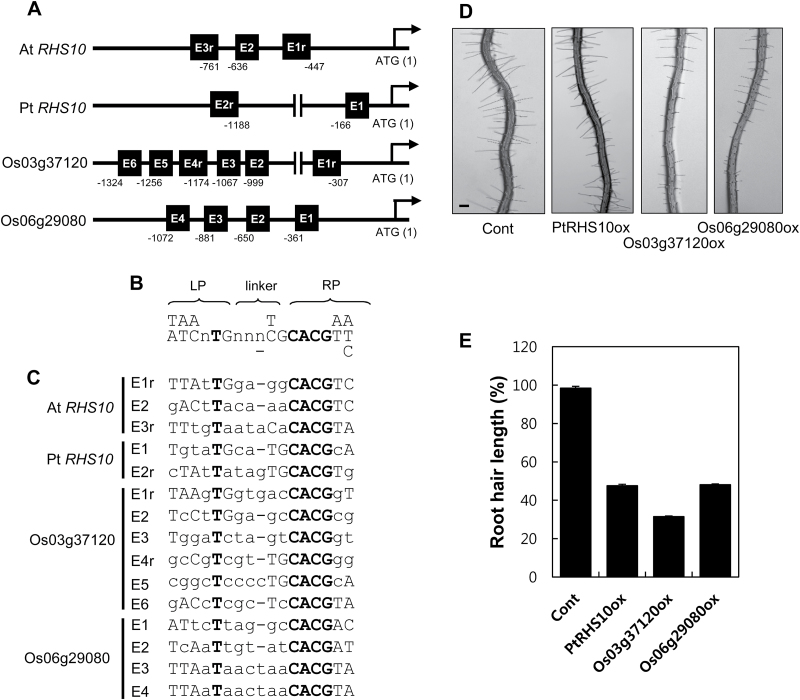
Functional analysis of RHS10 orthologs from rice and poplar in Arabidopsis root hair growth. (A) Locations of the putative root hair-specific *cis*-element (RHE) in the promoter region of Arabidopsis (At*RHS10*), poplar (Pt*RHS10*), and rice *RHS10* (Os03g37120 and Os06g29080). The RHE (black-boxed) position is indicated by the number relative to the start codon (ATG). An ‘r’ after the RHE number indicates a reverse orientation of the RHE. (B) The RHE consensus sequence. LP, left part; RP, right part. (C) The alignment of RHE sequences from *RHS10* orthologs. Abundant nucleotides are in upper case, and strictly conserved nucleotides are in bold. (D) Representative root images of the transformant lines with root hair specifically expressing poplar (PtRHS10ox, *ProE7:PtRHS10*) and rice *RHS10* (Os03g37120ox, *ProE7:Os03g37120*; Os06g29080ox, *ProE7:Os06g29080*) orthologs. Cont, control (*ProE7:YFP*). The scale bar is 100 μm in all images. (E) Root hair lengths of Arabidopsis transgenic lines with root hairs specifically expressing *RHS10* orthologs. Data represent the mean ±SE from 1996 (113 seedlings, Cont), 5686 (315 seedlings from 12 independent lines, PtRHS10ox), 4341 (311 seedlings from 10 independent lines, Os03g37120ox), and 4389 root hairs (313 seedlings from 13 independent lines, Os06g29080ox).

The At*RHS10* gene is expressed specifically in root hairs with at least three RHE motifs in the proximal promoter region (Fig. 4A; Won *et al.*, 2009). First, we examined whether *RHS10*-orthologous genes also include the RHE in their promoter regions. The promoters from rice and poplar orthologs included multiple RHEs; two RHEs for Pt*RHS10* and six or four RHEs for rice *RHS10* within 1400bp from the start codon (Fig. 4A). All RHE motifs from *RHS10*-orthologous sequences possessed functionally essential nucleotides such as ‘T’ on the left part (LP), ‘CACG’ on the right part (RP), and other positions with limited flexibility as defined by Kim *et al.* (2006) (Fig. 4B, C). This suggests that rice and poplar *RHS10*-orthologous genes could also be root hair specific and be genuine *RHS10* orthologs.

To test the orthologous relationship functionally, we introduced these orthologous sequences into the Arabidopsis root hair cell using *ProE7* and observed whether they also inhibit root hair growth. Both rice and poplar *RHS10* genes considerably inhibited root hair growth in Arabidopsis (53% for Pt*RHS10* and 52–69% for rice orthologs) (Fig. 4D, E). Although the strength of hair inhibition by these two orthologs was weaker than that by At*RHS10*, a result potentially due to different cell wall compositions or kinase activities among species, their root hair-inhibitory activities strongly suggest that RHS10 orthologs play a general role in root hair growth in angiosperms.

**Fig. 5.**
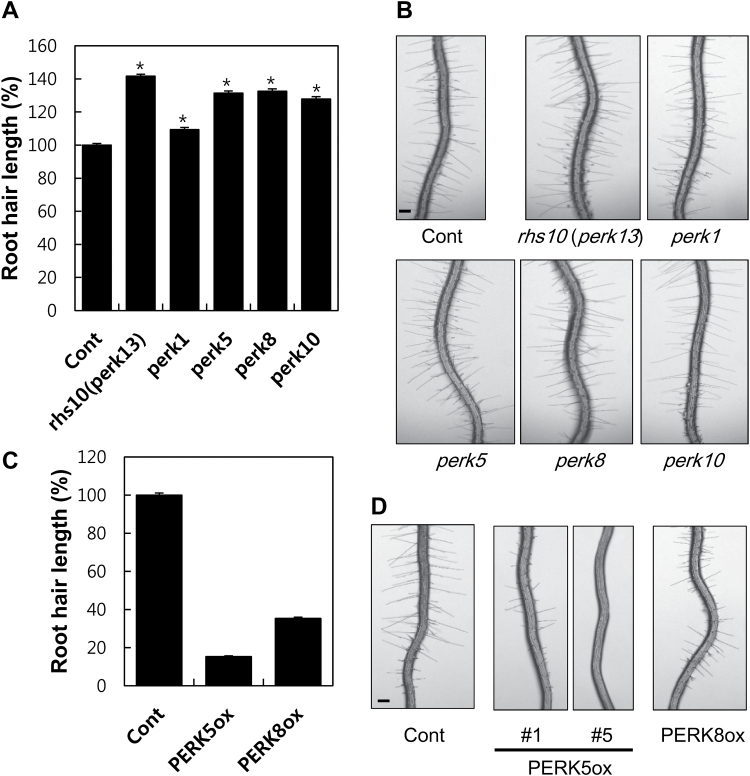
Root hair-inhibitory function of PERKs. (A) Root hair lengths of different *perk* mutants. Data represent the mean ±SE from 1504 [71 seedlings, Cont (Col-0)], 1201 (57 seedlings, *rhs10* or *perk13*), 1103 (53 seedlings, *perk1*), 992 (49 seedlings, *perk5*), 1148 (57 seedlings, *perk8*), and 768 (34 seedlings, *perk10*) root hairs. The values for *rhs10* (*perk13*), *perk1*, *perk5*, *perk8*, and *perk10* are significantly different (*P*<0.0001) from Cont values. (B) Representative root images of *perk* mutants. The scale bar is 100 μm in all images. (C) Root hair lengths of the transformants with root hairs specifically expressing *RHS10* paralogs. Data represent the mean ±SE from 1458 [81 seedlings, Cont (*ProE7:YFP*)], 6280 [348 seedlings from 13 independent lines, PERK5ox (*ProE7:PERK5*)], and 4374 [243 seedlings from eight independent lines, PERK8ox (*ProE7:PERK8*)] root hairs. (D) Representative root images of PERK overexpression lines. The scale bar is 100 μm in all images.

In addition to orthologs, we also tested if RHS10 paralogs have a root hair-inhibitory function because they share a similar protein structure. We chose four representative RHS10 paralogs from different Arabidopsis PERK clades (Supplementary Fig. S4), and analyzed root hair phenotypes of mutants and overexpression transformants. Loss-of-function mutants (Supplementary Fig. S7) for *PERK1*, *PERK5*, *PERK8*, and *PERK10* all showed significantly longer root hair phenotypes, whereas the phenotypes of *perk5*, *perk8*, and *perk10* were comparable with that of *rhs10* (*perk13*), and *perk1*’s phenotype was relatively weak (Fig. 5A, B). *ProE7*-driven overexpression of *PERK5* and *PERK8* greatly inhibited root hair growth (Fig. 5C, D). This result suggests that PERK family members transduce similar extracellular signals and target similar effectors, at least in the root hair cell.

### Auxin and ethylene cannot rescue RHS10-inhibited root hair growth

The root hairless phenotype of *rhd6* can be rescued by the addition of auxin or ethylene, suggesting that these two hormones act downstream of RHD6 (Masucci and Schiefelbein, 1994, 1996). To determine the regulatory hierarchy between these hormones and RHS10 for root hair growth, we tested whether RHS10-mediated root hair inhibition can be rescued by auxin and ethylene. The WT and *rhs10* mutants grew longer root hairs in response to 20nM indole-3-acetic acid (IAA) and 5 μM 1-aminocyclopropane-1-carboxylic acid (ACC; the ethylene precursor), whereas root hair defects of RHS10ox transformants could not be rescued by these two hormones (Supplementary Fig. S8A). A similar result was obtained with root hair-specific PERK8-overexpression transformants (Supplementary Fig. S8B). PERK8 shares a similar structure with RHS10/PERK13 and belongs to a neighboring clade (Supplementary Figs S4–S6). These results indicate that PERK family members may generally function as repressors of root hair growth downstream of hormonal action.

### Genetic interaction of RHS10 with other root hair genes

To characterize the relationship between *RHS10* and other root hair morphogenetic genes, double or triple mutants (or transformants) with *rhs10* were generated, and their root hair phenotypes were analyzed. We chose hormone- (*ProE7:axr2-1*, *ProE7:PIN3*, and *ein2*), signaling- (*rhd2-1* and *cow1*), and cell wall-related (*lrx1*, *lrx2*, and *rol1*) genes.

**Fig. 6.**
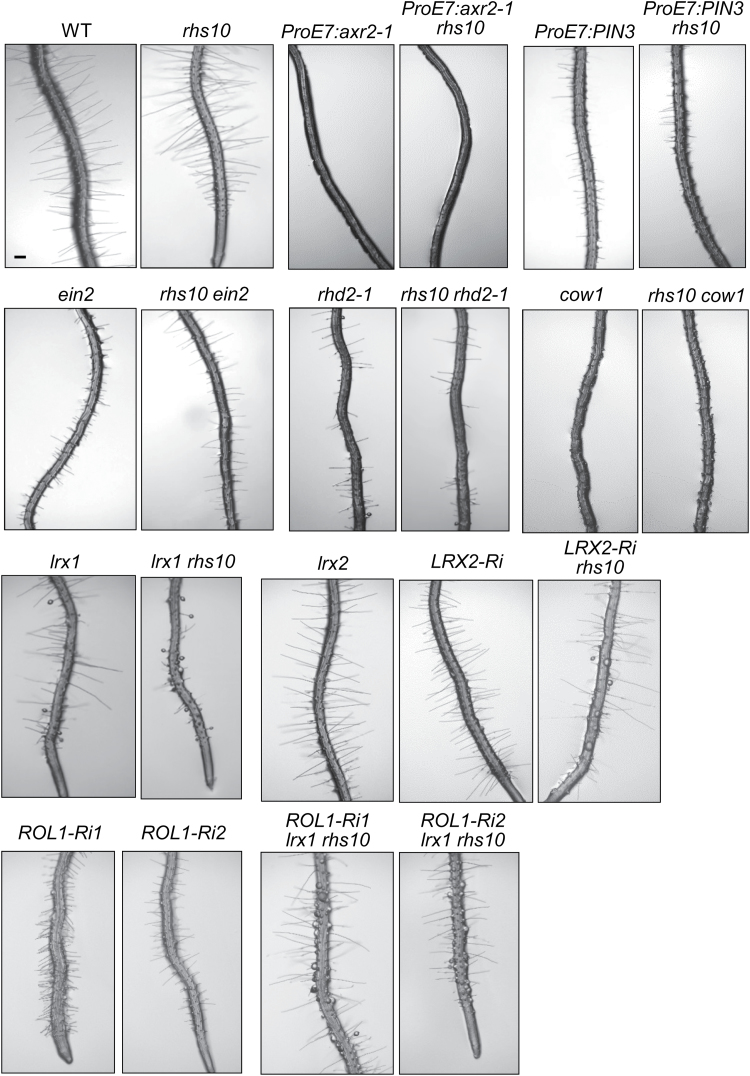
Genetic interactions of *RHS10* with other root hair-regulating genes. Root hair phenotypes of wild-type (WT), mutant, and transformant seedlings. The scale bar is 100 μm in all images.

Root hair-specific expression of *axr2-1* (*ProE7:axr2-1*), the dominant mutant of *IAA7*, in *rhs10* completely inhibited root hair growth (Fig. 6), most probably by inhibiting auxin signaling in the root hair cell. Root hair-specific overexpression of *PIN3* (*ProE7:PIN3*), the auxin efflux carrier, also greatly suppressed root hair growth of *rhs10* by depleting auxin in the root hair cell. These data indicate that auxin is necessary for root hair growth. In these transgenic backgrounds, the *rhs10* mutant has lost its capacity to grow longer hairs, resulting in the original transgenic phenotypes (suppressed root hair growth). The ethylene signaling-defective *ein2* mutant also grew shorter root hairs than the WT, and the *rhs10 ein2* double mutant also showed *ein2*-like short root hair phenotypes. Defects in *RHD2* (encoding a NADPH oxidase; Foreman *et al.*, 2003) and *COW1* (*CAN OF WORMS 1*; encoding a phosphatidylinositol phosphate transfer protein; Böhme *et al.*, 2004) also suppressed the enhanced root hair growth phenotype of *rhs10*. These results collectively suggest that these genes are epistatic to *rhs10* during root hair growth.

The cross lines of *rhs10* and several cell wall-related mutants showed interesting phenotypes. *LRX1* and *LRX2* encode leucine-rich repeat extensin proteins whose defects cause aborted, swollen, and/or branched root hair phenotypes (Baumberger et al., 2001, 2003; Fig. 6). The single *lrx2* mutant did not show any detectable root hair phenotype but synergistically functioned with the *lrx1* mutant (Baumberger *et al.*, 2003). In this study, we introduced double loss-of-function mutations in *LRX* genes and *RHS10* to determine their relationship. Because the genetic loci for *RHS10* and *LRX2* are close to each other on chromosome 1, we generated an RNAi line of *LRX2* (*Pro35S:LRX2-Ri*) to cross with *rhs10*. The *lrx1*-*rhs10* double mutant expressed more severe *lrx1* phenotypes (i.e. more aborted root hairs) than the single *lrx1* mutant (Fig. 6). Furthermore, *rhs10* in the *LRX2-Ri* background expressed the aborted root hair phenotype, although the *LRX2-Ri* transformant itself did not express such a phenotype (Fig. 6). These results indicate that the loss of *RHS10* enhances *LRX* defects in the root hair.

On the other hand, *ROL1* [repressor of *lrx1*, encoding a rhamnose (a pectin unit) biosynthetic enzyme] behaved in such a way that its loss in the *lrx1* background restored the normal root hair phenotype (Diet *et al.*, 2006). Because the genetic loci for *RHS10* and *ROL1* are close to each other on chromosome 1, we generated two independent RNAi lines of *ROL1* (*Pro35S:ROL1-Ri1* and *2*) and crossed them with *rhs10*. The suppression of *ROL1* shortened the root hair, and the *rhs10 lrx1 ROL1-Ri* triple mutant still harbored aborted root hairs, but showed restoration of root hair growth compared with the *lrx1 rhs10* double mutant or *ROL1-Ri* lines (Fig. 6), suggesting that suppression of *ROL1* can rescue the *lrx* mutants as previously reported (Diet *et al.*, 2006), even under the *rhs10* background. It is an intriguing that the *rhs10 lrx1 ROL1-Ri* triple mutant expresses a phenotype with longer root hairs than *ROL1-Ri* lines. The enhanced *lrx* root hair phenotypes caused by the loss of *RHS10* might result from overall increases to the hair-expanding capacity in the *rhs10* background, which should lead to the weakened root hair cell walls of *lrx* mutants breaking more readily.

### RHS10 suppresses ROS accumulation

**Fig. 7.**
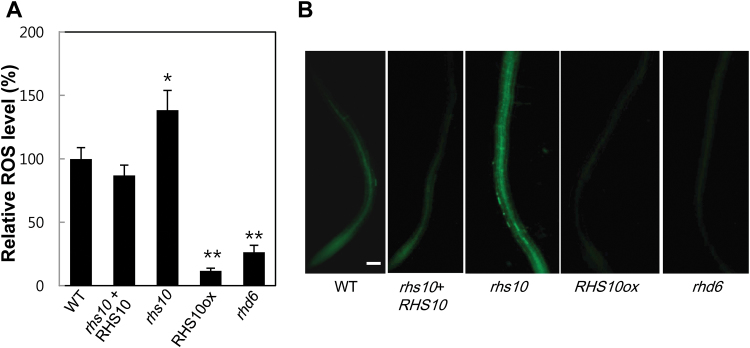
Measurement of ROS accumulation in the root. (A) Relative levels of ROS in different genotypes. *rhs10*+RHS10=*ProRHS10:RHS10* in *rhs10* and RHS10ox=*ProE7:RHS10*. Data represent the mean ±SE from 11–23 roots. The values are relative to the WT value (100%) and are significantly different from the WT value at *P*<0.05 (*) and *P*<0.01 (**). (B) Visualization of ROS in the primary roots of seedlings using H_2_DCFDA. The scale bar is 100 μm in all images.

ROS production by RHD2 is necessary for normal root hair growth (Foreman *et al.*, 2003). Here, we tested if RHS10 regulates ROS production to affect root hair growth. The ROS level in the root was visualized by H_2_DCFDA, an ROS probe. The long-haired *rhs10* mutant showed significantly higher ROS accumulation than the WT, and complementation of *rhs10* with *RHS10* (by its own promoter) lowered the ROS level almost to that of the WT (Fig. 7). Conversely, overexpression of *RHS10* under *ProE7* in the WT background (RHS10ox) considerably suppressed ROS accumulation, and hairless *rhd6* accumulated much fewer ROS than the WT (Fig. 7). This result demonstrates that RHS10-mediated regulation of root hair growth is correlated with the ROS level.

### RHS10 affects RNA contents probably by modulating RNS2

**Fig. 8.**
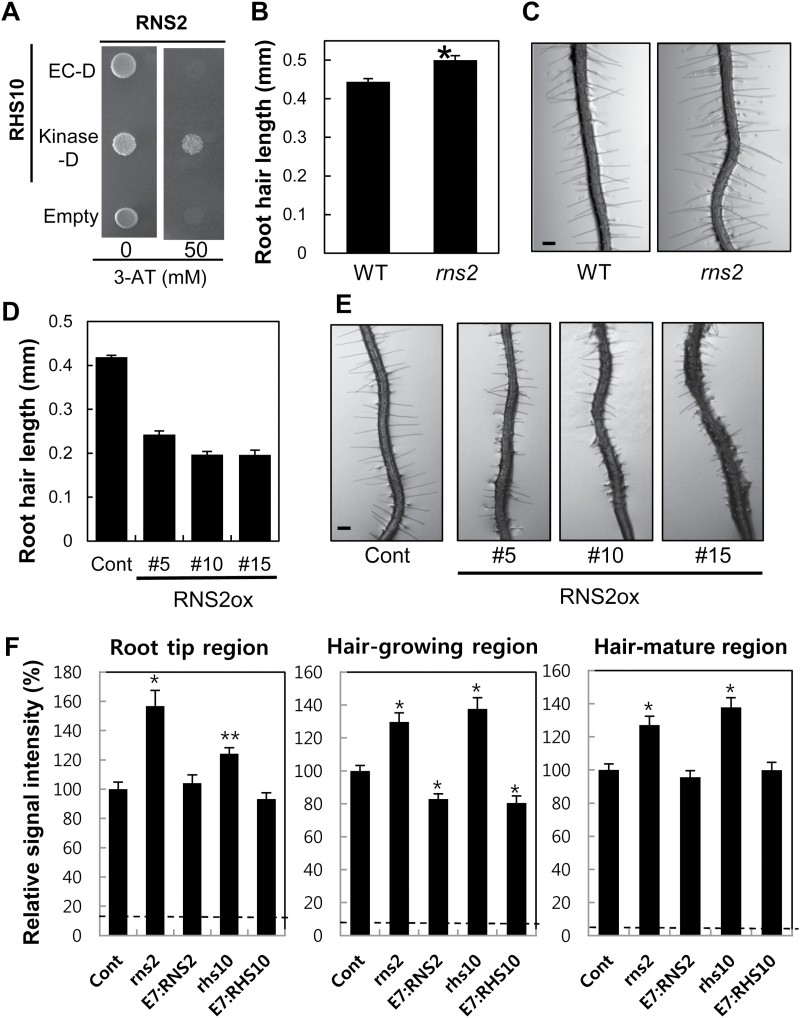
Functional analysis of the RHS10 downstream process. (A) Yeast two-hybrid analysis of the interaction between the extracellular (EC-D) or kinase domain (Kinase-D) of RHS10 and the whole RNS2 sequence. The EC-D includes fragments 1–235 and Kinase-D includes fragments 256–710 from the whole RHS10 sequence. ‘Empty’ is the vector control. (B) The loss-of-function RNS2 mutant grows slightly longer hairs than the wild type (WT). Root hair length was estimated with a total of 98–100 root hairs from 10 seedlings of the WT and *rns2* mutant. Data represent the mean ±SE The value of *rns2* is significantly (*P*<0.0001) different from the WT value. (C) Representative root images of WT and *rns2* mutant seedlings. The scale bar is 100 μm in all images. (D) RNS2 overexpression inhibits root hair growth. Root hair length was estimated with a total of 105–717 root hairs from 11–74 seedlings from the control (Cont, *ProE7:YFP*) and independent RNS2 overexpression lines (RNS2ox, *ProE7:RNS2*). (E) Representative root images of Cont and RNS2ox transformant lines. The scale bar is 100 μm in all images. (F) Relative RNA contents from each root developmental zone of the control (Cont, *ProE7:YFP*), mutant (*rns2* and *rhs10*), and transformant (E7:RHS2=*ProE7:RHS2*, E7:RHS10=*ProE7:RHS10*) seedlings. Data represent the mean ±SE from 47–49 seedlings. Differences are significant at *P*<0.0005 (*). Broken lines indicate fluorescence levels without SYTO dye.

In order to identify the direct molecular targets of RHS10, we performed a Y2H screening. We obtained 79 positive clones from the screening and focused on one gene called *RIBONUCLEASE 2* (*RNS2*, AT2G39780). Four (two identical) out of the 79 positive clones represented *RNS2*, all of which included most of the *RNS2* coding region; 86–963, 85–1024, and 95–1018bp of the 1074-bp full-length cDNA sequence (start to stop codon). In a repeated Y2H assay, RNS2 interacted with the C-terminal kinase domain but not with the N-terminal ECD of RHS10 (Fig. 8A).

**Fig. 9.**
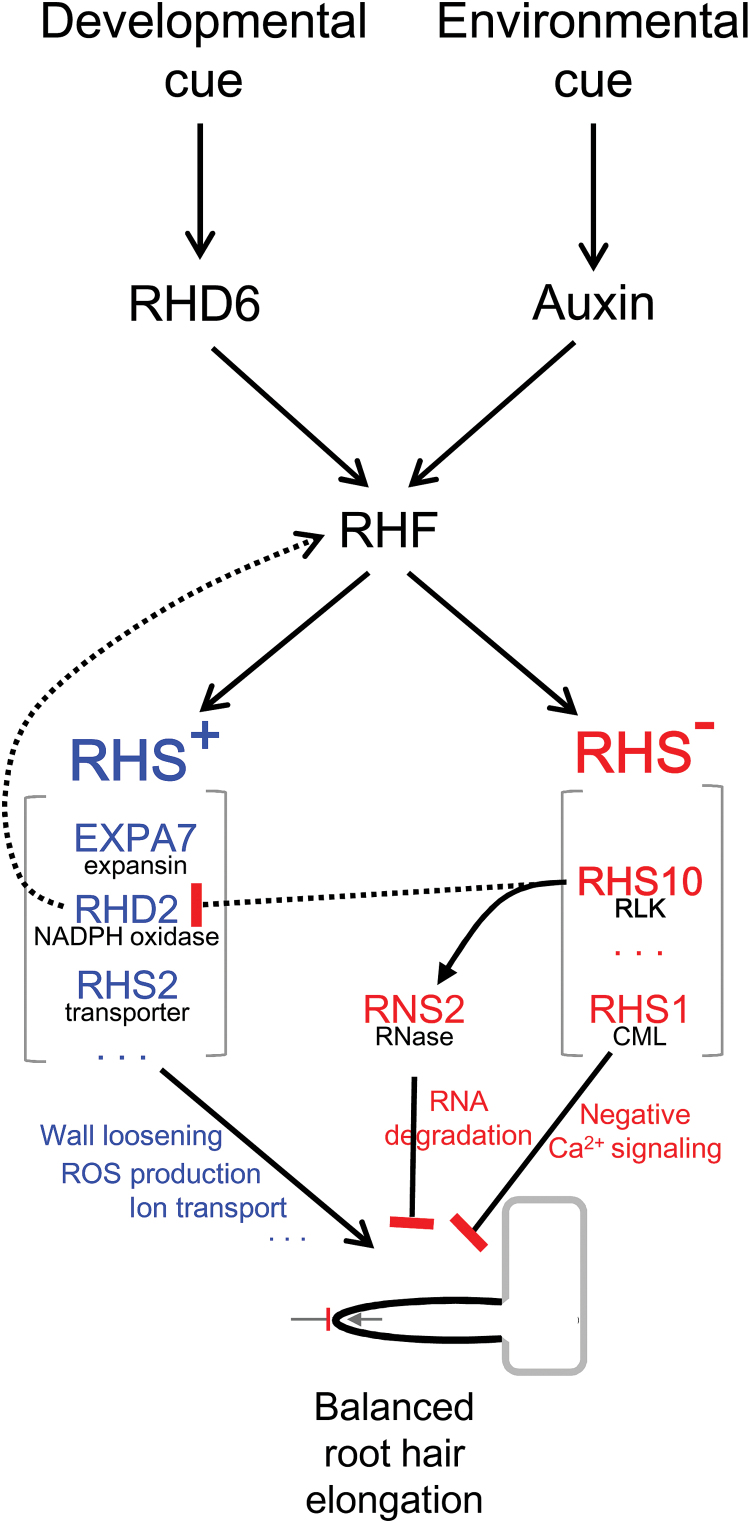
A model illustrating balanced root hair tip growth. RHE-containing root hair-specific (RHS) genes are under the control of the putative root hair-specific transcription factor (RHF). *RHS* genes are divided into two subgroups, where *RHS* ^+^ (blue) enhances, but *RHS* ^*−*^ (red) inhibits root hair elongation. RHS10, a proline-rich RLK, may play a key negative role in root hair elongation by the global control of RNA degradation via RNS2, an RNase. On the other hand, RHS1, a calmodulin-like (CML) protein, may negatively affect root hair elongation through an unknown negative Ca^2+^ signaling pathway. The balanced activities between RHS^+^ and RHS^*−*^ will generate a specific root hair size under certain cellular and environmental conditions. Blunt end, negative action; arrow, positive action; solid line, experimentally supported; broken line, hypothetical.

Next, we examined whether RNS2 modulates root hair growth in line with RHS10 function. A loss-of-function *rns2* (SALK_069588; Supplementary Fig. S7) null mutant (Hillwig *et al.*, 2011) and root hair-specific *RNS2* overexpression lines (*ProE7:RNS2*, RNS2ox) were observed for their root hair phenotypes. The *rns2* mutant seedlings grew slightly longer (14%) root hairs compared with the WT (Fig. 8B, C). Conversely, RNS2ox lines grew much shorter root hairs, ~50% the length of the WT (Fig. 8D, E).

If RHS10 positively regulates RNS2, RHS10 would work positively to degrade RNA. To test this idea, we examined cellular RNA levels along regions of root development, root tips, root hair growing, and root hair maturation. To stain RNA in the root tissue, SYTO RNASelect Green Fluorescent Cell Stain (Hillwig *et al.*, 2011) was used, and fluorescence intensity was quantified to compare RNA levels of different genotypes. In all root regions, RNA levels were considerably increased in the *rns2* mutant background by 57, 30, and 27% compared with the control, in tips and growing and maturation regions of root hairs (Fig. 8F). Regarding the *rhs10* mutant, although a significant increase (24%) in RNA content was observed in the root tip region, a greater increase (~38%) occurred in growing and maturation regions. Conversely, root hairs specifically overexpressing *RHS10* (*ProE7:RHS10*) and *RNS2* (*ProE7:RNS2*) RNA significantly decreased but only in the hair-growing region. These results imply that RHS10 inhibits root hair growth at least by modulating RNS2-mediated RNA degradation.

Next, we tested whether the RHS10 kinase domain can phosphorylate RNS2. Heterologously expressed GST-fused proteins were used in the kinase assay. The fusion proteins were detected at their expected molecular sizes in the protein blot analysis using anti-GST antibody (Supplementary Fig. S9A). In the kinase assay including [γ-^32^P]ATP as a phosphate source, the RHS10 kinase domain did not appear to phosphorylate RNS2, whereas the kinase was autophosphorylated (Supplementary Fig. S9B). According to the deletion analysis of the RHS10 ECD, the D5 deletion, which includes only the kinase domain, failed to inhibit root hair growth (Fig. 3C). These results, when analyzed together, suggest that the RHS10 kinase domain is not able to target the downstream effector to inhibit root hair growth when acting alone, although it is capable of autophosphorylation (Supplementary Fig. S10).

## Discussion

The molecular function of PERKs has been poorly understood. In this study, by analyzing the molecular function of RHS10 and its PERK homologs in root hair growth, we infer their putative cell wall association motif, evolutionary conservation, and downstream mechanism.

### RHS10-mediated root hair inhibition requires arabinogalactan protein-like motifs in its ECD

The deletion analysis of the ECD of RHS10 revealed that only 47 amino acid residues (of 236 residues for the whole ECD) were sufficient to cause considerable RHS10-mediated root hair inhibition (Fig. 3). This is comparable with the result from serial deletion analysis of LRX1 extensin motifs, where deleting most extensin did not affect LRX function (Ringli, 2010). The short stretch of the RHS10 ECD region includes eight proline residues. Because proline residues are critical for the function of hydroxyproline-rich glycoproteins (HRGPs), this small number of proline residues may play a key role for the RHS10 ECD, which would mediate some extracellular signals, thereby allowing the RHS10 kinase domain to trigger the downstream root hair-inhibitory processes.

HRGPs can be divided into three subfamilies: extensins, arabinogalactan proteins (AGPs), and proline-rich proteins (PRPs) (Showalter *et al.*, 2010). These HRGPs have the characteristic repetitive motifs of SPx (x≥3) for extensins, PA/AP/SP/TP for AGPs, and KKPCPP/PVX(K/T)/PPV for PRPs, in which proline residues are subject to hydroxylation by prolyl hydroxylases and subsequent *O*-glycosylation (Showalter *et al.*, 2010). HRGP subfamilies have distinctive molecular and biological functions; for example, extensins aid in cell wall stiffening, and AGPs are used for extracellular signaling as well as cell wall integrity (Cassab, 1998; Humphrey *et al.*, 2007; Showalter *et al.*, 2010).

In terms of motifs, the ECD of PERK members includes not only extensin-like motifs, but also AGP motifs. RHS10 orthologs and paralogs from rice, poplar, Arabidopsis, and even *Physcomitrella* all have multiple (28–42) AGP motifs (Fig. 3A; Supplementary Fig. S6). In this context, the ECD of RHS10 orthologs and other PERKs can be considered as hybrid HRGPs of extensins and AGPs. Although the ECD of PERKs carry extensin-like SPx motifs, their ECD region almost completely lacks the YXY/VYK motif or even a tyrosine residue (Fig. 3A; Supplementary Fig. S6), which functions for peroxidase-mediated inter- and intramolecular cross-linking in canonical extensins (Epstein and Lamport, 1984; Lamport *et al.*, 2011). Interestingly, the tyrosine residue in the extensin part of LRX1 was also reported not to affect cell wall cross-links (Ringli, 2010). The weak extensin nature of the PERK ECD suggests that the PERK ECD may behave like AGPs at the molecular level. The nature of AGP for the function of the PERK ECD can be conceived by our result demonstrating that RHS10-mediated root hair inhibition required the minimal AGP motif-containing ECD region (D3-1 deletion), but not the extensin-like motif (Fig. 3). A recent study has demonstrated that some root hair-specific prolyl 4-hydroxylases are required for normal root hair growth (Velasquez *et al.*, 2011). Prolyl 4-hydroxylases hydroxylate the proline residues of extensins and AGPs, which will subsequently be glycosylated (Showalter *et al.*, 2010). It would be interesting to determine whether these root hair-specific prolyl 4-hydroxylases target proline residues of the RHS10 ECD.

Glycophosphatidylinositol (GPI)-anchored AGPs can reside in the PM, and some AGPs were shown to bind to pectins (Iraki *et al.*, 1989; Serpe and Nothnagel, 1995), indicating the possibility that AGPs act as sensors for cell wall dynamics (Humphrey *et al.*, 2007). WAKs (cell wall receptors) were shown to bind directly to pectins (Wagner and Kohorn, 2001), and PERK4 seems to associate with pectins (Bai *et al.*, 2009a). The loss of *RHS10* restored root hair growth, which was inhibited by loss of *ROL1* in the *lrx* background (Fig. 6), implying that the function of RHS10 requires a ROL1-mediated process for pectin organization. These results prompted us to hypothesize that the AGP motifs of the RHS10 ECD associate with certain cell wall components, causing the RHS10 kinase domain to transduce signals from wall structure changes into the cytoplasm. Cell wall-loosening proteins, such as expansins, can cause changes in cell wall structure (Cosgrove, 2000; Choi *et al.*, 2006), which then might be sensed by wall-associated kinases including WAKs and PERKs. Because root hair-specific expansins (Cho and Cosgrove, 2002; Kim *et al.*, 2006; Won *et al.*, 2009, 2010; Lin *et al.*, 2011; Yu *et al.*, 2011) and PERKs (this study) have been functionally well conserved in angiosperms, root hair growth would provide a model system to characterize the relationship between cell wall dynamics and PERK functions.

In terms of motif organization and molecular function, the ECD of PERKs is likely to be conserved in land plants. As mentioned previously, extensin-like and AGP motifs are conserved in RHS10 homologs from Arabidopsis, rice, and poplar, which commonly exhibit root hair-inhibitory effects, even in a moss homolog (Figs 4, 5; Supplementary Fig. S6). This implies that AGP motifs could be commonly operational for the molecular function of PERKs in land plants.

### RHS10-mediated signaling for root hair tip growth

We have a paucity of evidence regarding downstream targets of RLKs. There is no downstream process identified for PERKs. In this study, we proposed a downstream process of RHS10 action. In an effort to identify the direct interactor of RHS10 using a Y2H screening, an RNase (RNS2) was identified as an RHS10 interactor (Fig. 8A). Although phosphorylation of RNS2 by the RHS10 kinase domain was not observed *in vitro* (Supplementary Fig. S9), RNS2 displayed the root hair-related function in accordance with RHS10 function; namely, negative effects on root hair growth (Fig. 8B–E). Furthermore, the loss of RHS10 increased, and RHS10 overexpression decreased the level of RNA in the root hair-growing region of the root in co-ordination with RNS2 activity (Fig. 8F). These consistent phenotypic effects of RHS10 and RNS2 on root hair growth and RNA levels suggest that RNS2 is one of the downstream targets of RHS10. Recently, Humphrey *et al.* (2015) showed that the kinase domain of PERKs, including RHS10/PERK13, interacts with AGC family protein kinases. These results together suggest that PERKs have multiple targets to modulate cellular processes. It would be interesting to know if the RHS10 targets are cell-type specific.

Although the RHS10 kinase domain alone is able to phosphorylate itself, full or modulated kinase activity may require its ECD. The cell wall association and signaling through the RHS10 ECD might be the key modulating process for RHS10 to phosphorylate downstream targets and inhibit root hair growth. Considering the ECD deletion result, a short stretch of the ECD might be enough to perform this activity. The failure of root hair inhibition by the D5 deletion of RHS10, which includes only the kinase domain, also supports the idea that the ECD is necessary to phosphorylate downstream targets (Supplementary Fig. S10); however, we do not exclude other possibilities, such that the mislocalization of the RHS10 kinase domain, owing to the loss of a TM domain, results in failure to identify proper targets.

RNS2 belongs to class II RNase T2, whose molecular and biological roles have rarely been characterized (MacIntosh *et al.*, 2010). Because RNS2 is highly expressed throughout diverse tissues, it was suggested to play a housekeeping role by recycling RNA (Taylor *et al.*, 1993; Bariola *et al.*, 1999; Hillwig *et al.*, 2011). RNS2 activity has neutral pH optima and is very low under acidic conditions (Hillwig *et al.*, 2011). Although RNS2 was known to localize mainly to the endoplasmic reticulum and vacuoles, where the pH is acidic, a minor portion of RNS2 appears to be present in the cytosol where pH is neutral, many RNA substrates exist, and RNS2 activity is optimal (Hillwig *et al.*, 2011). PM-localized RHS10 might target this cytosolic RNS2. While rRNAs are the most prominent substrates owing to their abundance in the cell, there is a possibility that RNS2 also targets other RNA species (Hillwig *et al.*, 2011). In addition to its role in cellular homeostasis by its RNA-recycling activity (Hillwig *et al.*, 2011), RNS2 may degrade RNAs that are required for various cellular functions. In root hair cells, an overall decrease in the cellular RNA level should be inhibitory to root hair growth (Fig. 8).

It is conceivable that RHS10 has multiple targets. Although the mechanistic process has yet to be characterized, RHS10 obviously showed an inhibitory effect on ROS accumulation in the root (Fig. 7). RHD2 (an NADPH oxidase)-mediated ROS generation is required to maintain root hair tip growth, most probably by modulating Ca^2+^-mediated vesicle trafficking toward the growing hair tip (Foreman *et al.*, 2003). Including *RHS10*, *RHS* genes are positively regulated by RHD2 (Jones *et al.*, 2006; Won *et al.*, 2009), suggesting that ROS not only affect the accumulation of materials for hair tip growth, but also modulate the production of protein tools for root hair growth. Whereas RHD2 regulates downstream *RHS* genes, the *RHD2* gene itself is likely to be regulated by an unidentified RHE-binding transcription factor (RHF; Kim *et al.*, 2006) since its proximal promoter region includes an RHE (Won *et al.*, 2009; Cho, 2013), and its expression in the root epidermis is hair cell specific (Brady *et al.*, 2007). Overall, these relationships would lead to feedback and feed-forward pathways among RHF, RHD2, and RHS10, as shown in Supplementary Fig. S11, which will result in balanced root hair elongation.

### Positive versus negative players in root hair elongation

The function of RHS10, together with RHS1, in root hair development is unique in that it negatively regulates root hair tip growth. Although the loss of *AKT1* (encoding a potassium transporter), *IPK2α* (encoding an inositol polyphosphate kinase), or *SIZ1* (encoding a SUMO E3 ligase) results in longer root hair growth, this occurs only when potassium, calcium, or phosphate are deficient, respectively (Desbrosses *et al.*, 2003; Miura *et al.*, 2005; Xu *et al.*, 2005). In addition to *RHS10*, the loss of *RHS1* (encoding a calmodulin-like protein) also enhances, and its overexpression inhibits, root hair elongation (Won *et al.*, 2009). While numerous genes positively contribute to root hair elongation without affecting hair morphology, such as branching, waving, and swelling (Grierson and Schiefelbein, 2008; Won *et al.*, 2009), these two *RHS* gene products are, so far, the only known negative players in root hair elongation under normal conditions, except for the proteins that negatively regulate cellular auxin activity (Grierson and Schiefelbein, 2008).

Among the known RHE-containing root hair-specific genes, *RHD2* (Schiefelbein and Somerville, 1990), *RHS2* (Won *et al.*, 2009), and *EXPA7* (Lin *et al.*, 2011) have been shown to affect root hair elongation positively in Arabidopsis. Therefore, in terms of their effect on root hair elongation, *RHS* genes can be divided into two subgroups: root hair-enhancing *RHS* (*RHS* ^*+*^) and root hair-inhibiting *RHS* (*RHS* ^*−*^) (Supplementary Fig. S11). *RHS* genes are thought to be controlled by RHF, which appears to be conserved, at least in the angiosperm lineage (Kim *et al.*, 2006). Because there is no noticeable structural difference in the RHE consensus sequence between *RHS* ^*+*^ and *RHS* ^*−*^ (Won *et al.*, 2009), these two *RHS* subgroups are likely to be equally controlled by RHF. This suggests that both positive and negative *RHS* genes would be equally required for normal root hair elongation, leading to the hypothesis that diverse environmental factors affecting the final root hair length might directly regulate either RHS^+^ or RHS^*−*^ proteins rather than their transcription.

## Supplementary data

Supplementary data are available at *JXB* online.

Figure S1. Root hair phenotypes of different insertion mutant lines of RHS10.

Figure S2. The effect of RHS10:GFP fusion protein overexpression on root hair growth.

Figure S3. Confocal microscopic images of PIN3:GFP in a root hair cell after plasmolysis.

Figure S4. Phylogenetic relationship of PERKs.

Figure S5. Alignment of RHS10 homologs.

Figure S6. The N-terminal protein structure of RHS10 homologs.

Figure S7. T-DNA insertion positions in *rns2* and *perk* mutants.

Figure S8. Effects of auxin and an ethylene precursor on root hair restoration of RHS10ox or PERK8ox transformants.

Figure S9. Protein blot analysis and an *in vitro* kinase assay of RNS2 and the RHS10 kinase domain.

Figure S10. A model for the role of the extracellular domain (ECD) of RHS10.

Table S1. Primer list.

Supplementary methods.

Supplementary Data
