# Supplementary material for: Cell wall-associated ROOT HAIR SPECIFIC 10, a proline-rich receptor-like kinase, is a negative modulator of Arabidopsis root hair growth
Source: J Exp Bot. 2016 Feb 16;67(6):2007–22. doi: 10.1093/jxb/erw031 (PMC4783376; doi:10.1093/jxb/erw031)
Supplement: Supplementary Data [file supp_67_6_2007__index.html]

Cell wall-associated ROOT HAIR SPECIFIC 10, a proline-rich receptor-like kinase, is a negative modulator of Arabidopsis root hair growth — Cell wall-associated ROOT HAIR SPECIFIC 10, a proline-rich receptor-like kinase, is a negative modulator of Arabidopsis root hair growth — Supplementary Data 

# Cell wall-associated ROOT HAIR SPECIFIC 10, a proline-rich receptor-like kinase, is a negative modulator of Arabidopsis root hair growth

## Supplementary Data

Data files

- supplementary\_figures\_S1\_S10\_table\_S1\_methods.pdf - Supplementary Data
