# Supplementary material for: Cell wall-associated ROOT HAIR SPECIFIC 10, a proline-rich receptor-like kinase, is a negative modulator of Arabidopsis root hair growth
Source: J Exp Bot. 2016 Feb 16;67(6):2007–22. doi: 10.1093/jxb/erw031 (PMC4783376; doi:10.1093/jxb/erw031)
Supplement: Supplementary Data [file supp_erw031_supplementary_figures_S1_S10_table_S1_methods.pdf]

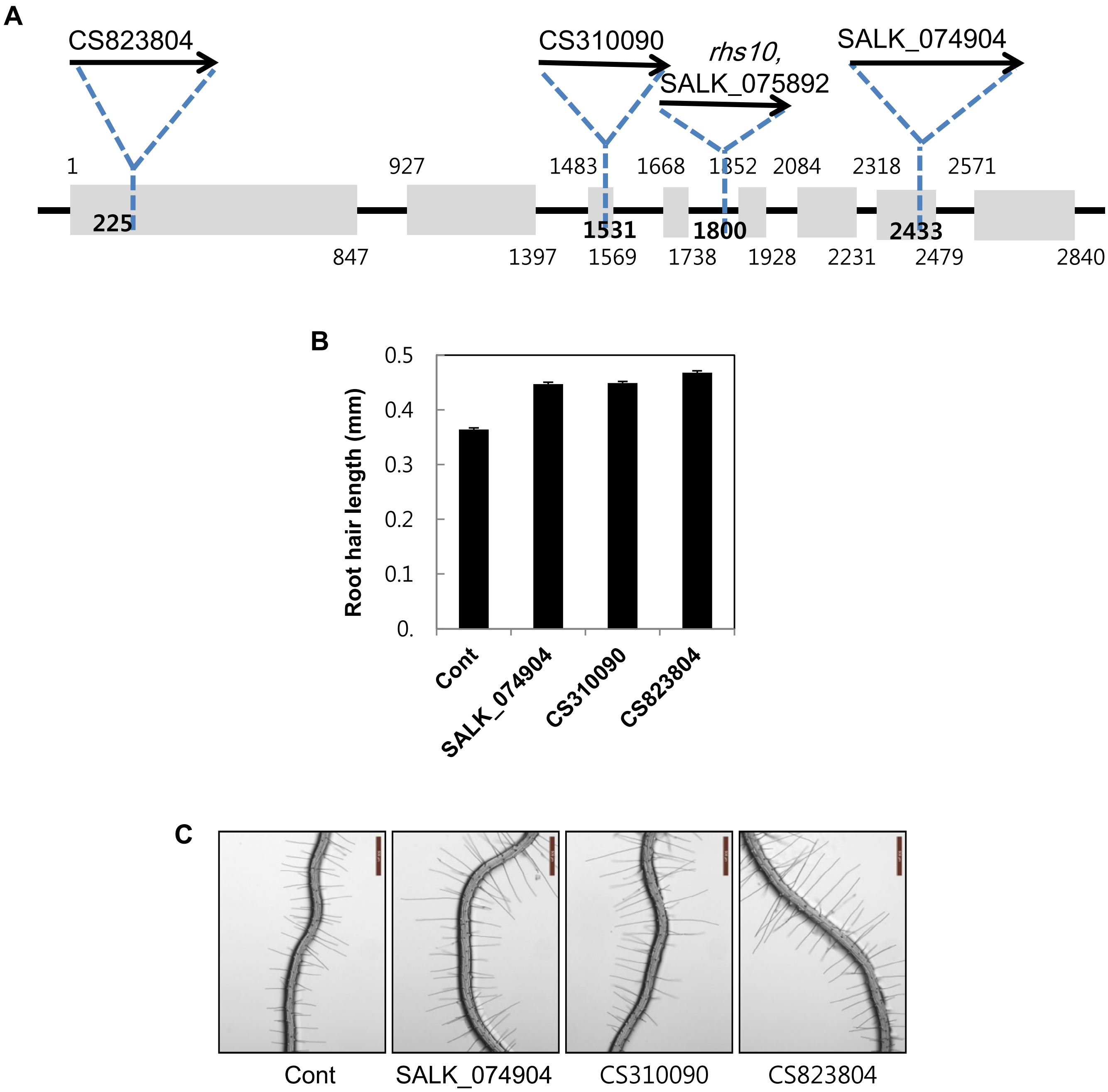

Fig. S1. Root hair phenotypes of different insertion mutant lines of RHS10. (A) A schematic diagram showing T-DNA insertion sites in the RHS10 gene. Exons and introns are indicated by gray boxes and black lines, respectively. (B) Root hair lengths of Cont and mutants lines. Data represent means±s.e. from 955~1181 root hairs (from 29~37 seedlings). (C) Representative root images of control (Cont; Col-0), loss-of-function *rhs10* mutants alleles (SALK_074904, CS823804, CS823804). Bar is 500 µm for all.

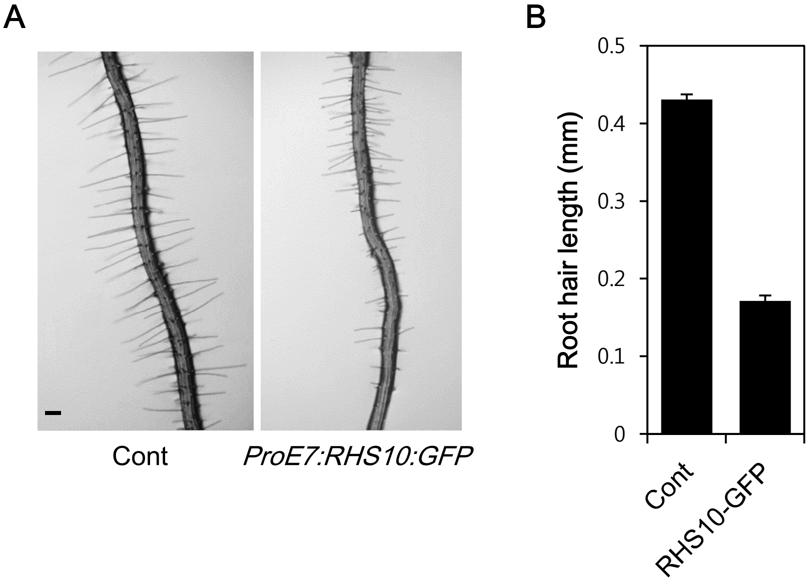

Fig. S2. The effect of the RHS10:GFP fusion protein overexpression on root hair growth. (A) Representative root images of control (Cont; *ProE7:YFP*) and root hair-specific RHS10:GFP expression line (*ProE7:RHS10:GFP*). Bar is 100 µm for all. (B) Root hair lengths of Cont and RHS10:GFP line. Data represent means±se from 235~260 root hairs (from 24~26 seedlings).

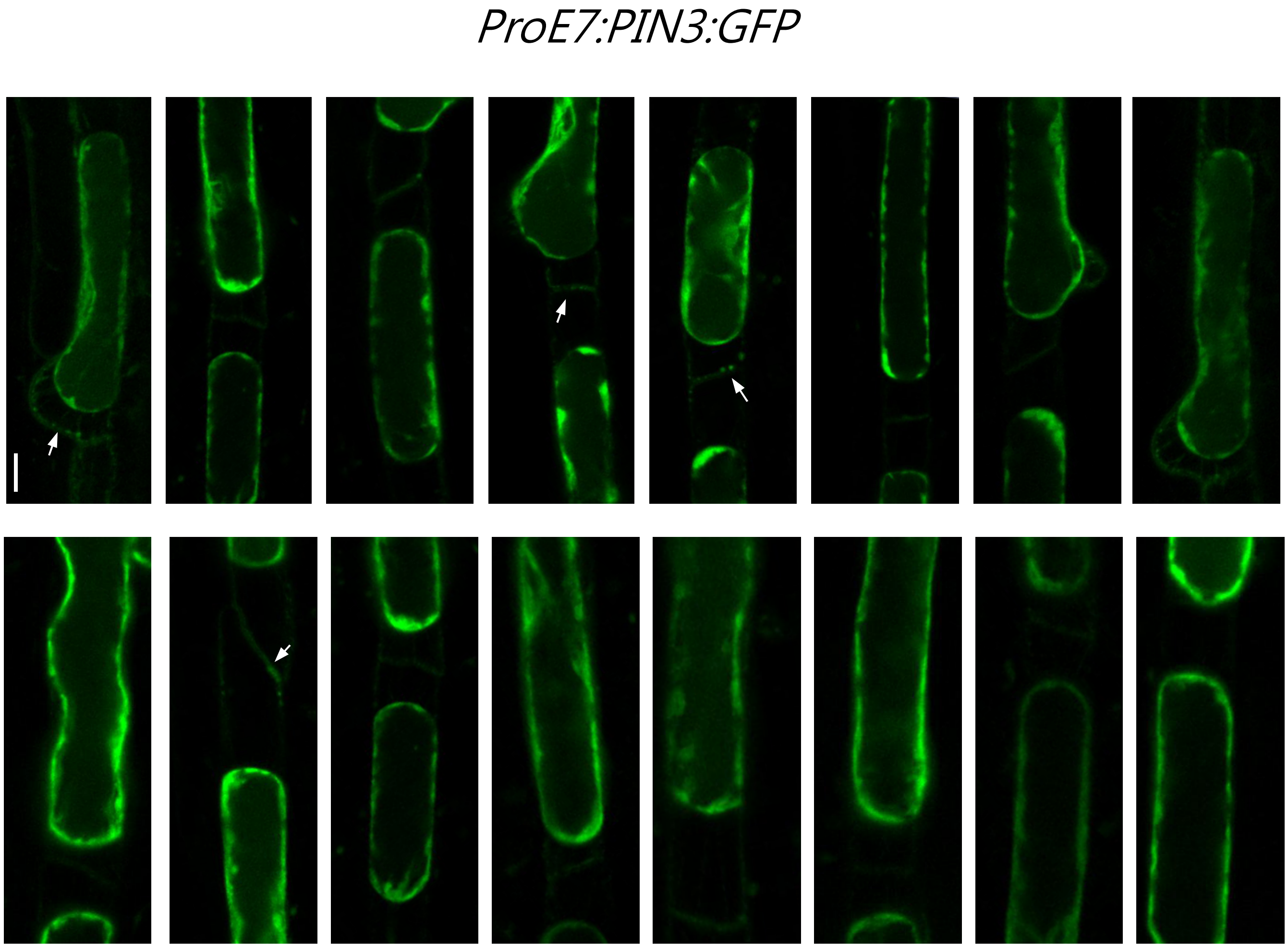

Fig. S3. Confocal microscopic images of PIN3:GFP in the root hair cell after plasmolysis. *ProE7:PIN3:GFP* transgenic seedling roots were plasmolyzed by 0.45 M mannitol and PIN3:GFP signals were observed under a confocal microscopy. Although some PIN3:GFP signals were left in the cell wall after plasmolysis (arrows), there were less frequent and weaker than RHS10:GFP signals.. Bar is 10 μm for all.

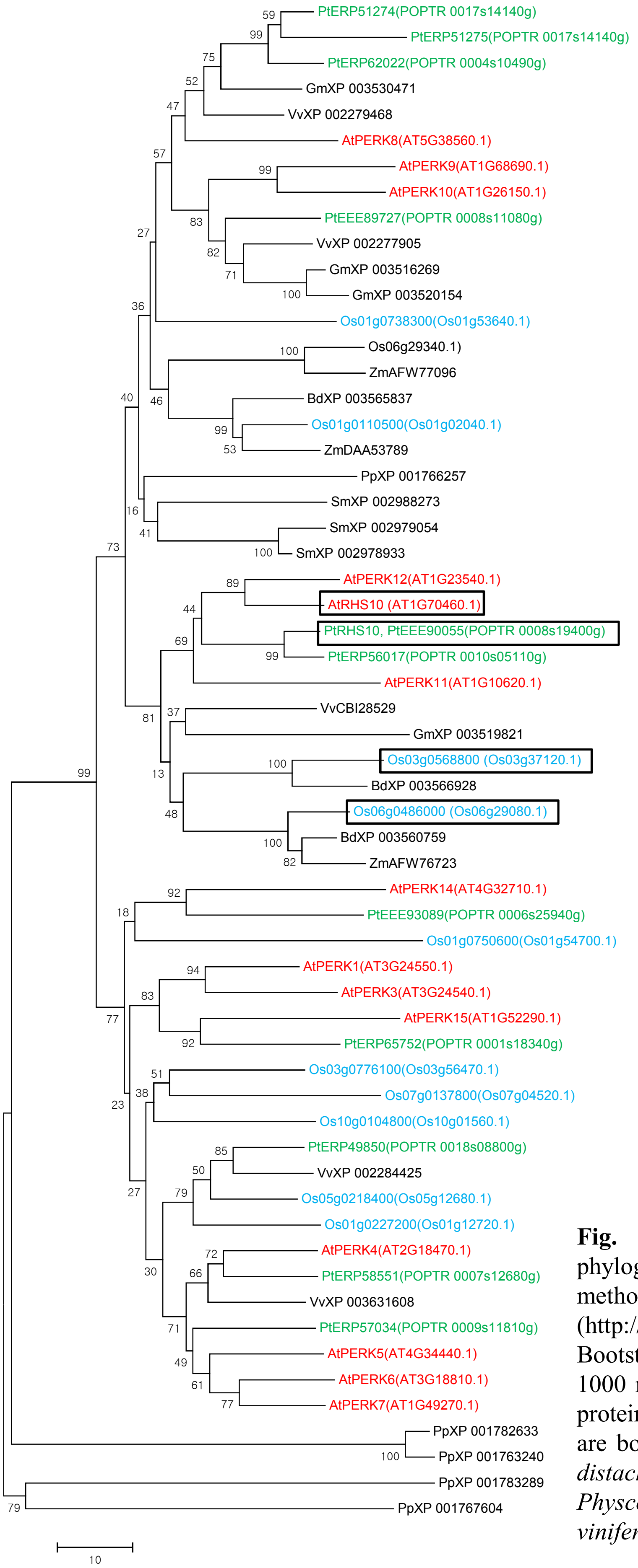

Fig. S4. Phylogenetic relationship of PERKs. The phylogenetic analysis was done by the Neighbor-Joining method using the MEGA4 software package (http://www.megasoftware.net/mega4/mega.html). Bootstrap values are given at the nodes as a percentage of 1000 replicates. *Arabidopsis* RHS10 and its orthologous proteins from rice and poplar, which were studied here, are boxed. At, *Arabidopsis thaliana*; Bd, *Brachypodium distachyon*; Gm, *Glycine max*; Os, *Oryza sativa*; Pp, *Physcomitrella patens*; Pt, *Populus trichocarpa*; Vv, *Vitis vinifera*; Zm, *Zea mays*

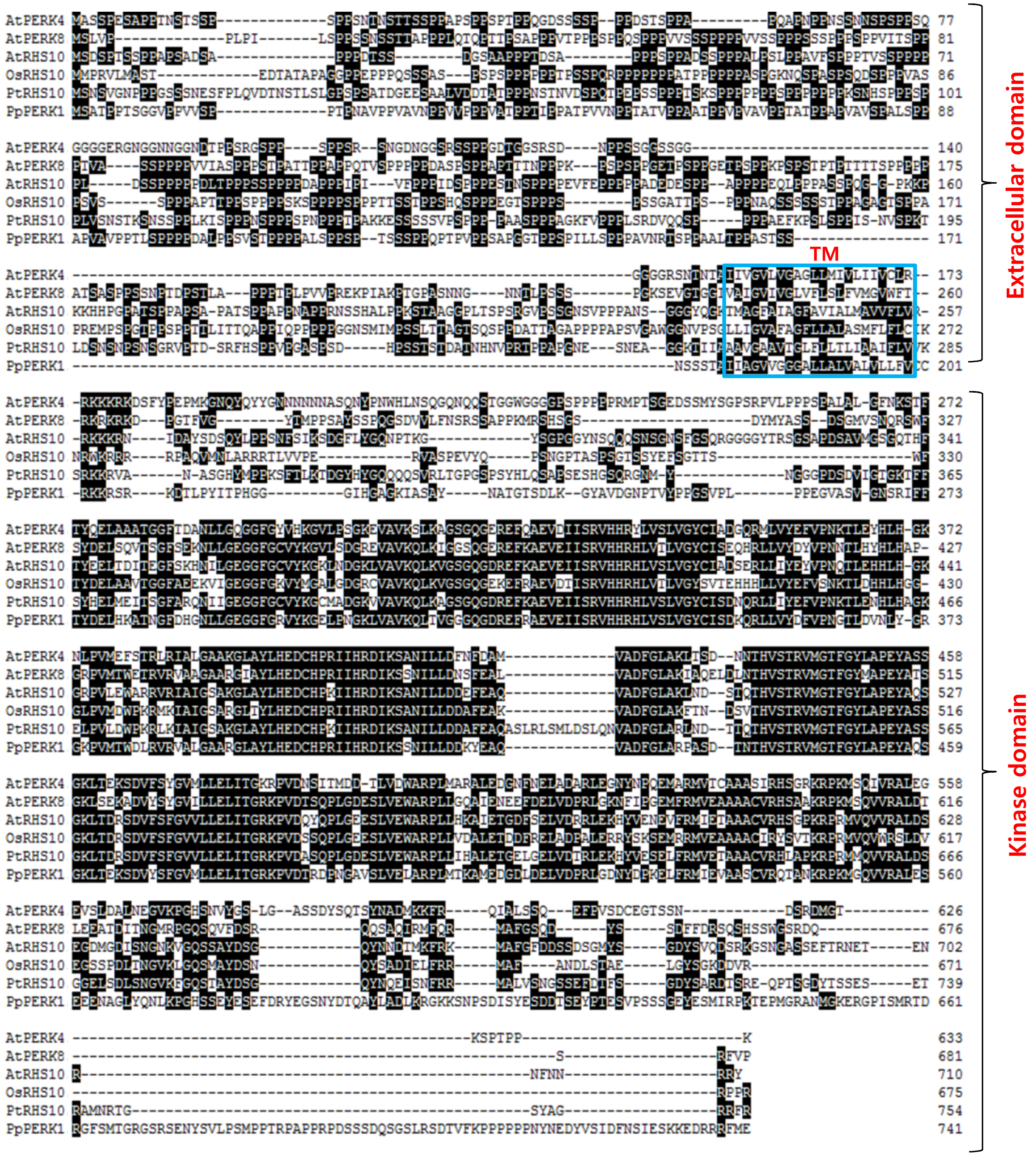

Fig. S5. Alignment of RHS10 homologs. The alignment was done by Clustal V. The trans-membrane (TM) domain was predicted by TMHMM Server v. 2.0 (http://www.cbs.dtu.dk/services/TMHMM-2.0/)

Os03g0568800(Os03g37120.1) (42 AGP motifs)

MMPRVLMAST EDTATAPAGG PPEPPPQSSS ASPSPSPPPP PPTPSSPQRP
PPPPPPATPP PPPPASPGKN QSPASPSQDS PPPVASPSVS SPPPAPTTPP
SPPPPSKSPP PPSPPPTTSS TPPSHQSPPE EGTSPPPSPS SGATTPSPPP
NAQSSSSSST PPAGAGTSPP APREMPSPGT PPSPPTTLIT TQAPPIQPPP
PPGGNSMIMP SSLTTAGTSQ SPPDATTAGA PPPPAPSVGA WGGNVPSGLL
IGVAFAGFLL ALASMFLFLC IKNRWKRRRR PAQVMNLARR RTLVVPERVA
SPEVYQPSNG PTASPSGTSS .....→ continued to the kinase domain

Os06g0486000(Os06g29080.1) (21 AGP motifs)

MSSESDQAPS PSSPSSSSSS SSGNGSNKAP PPEESDNSSS NGSSSSSPTP
PSSQSSDSDS GGGSSSPSQG SSSPSPPPPS GSSSESHSSP PPAPPQSSSS
SSSSSSSGGG SKSSPEAPSP PSESSGNGGG GGGGGRSSPP PNWSPPPQQQ
QQHQSGGSTP SPPPSPSSNQ PPPSSGSSAS SSEPSPPRSS PPPSPPQSSG
GNNGQPPKPS GGQQQAPPQS PPSAANQSVV FIPVPVASNS PPGMLPPPQV
IDATPSGAIS STNFPGGRNS TAGSSNTSLS QQQHTTVSST AQASSSGHIA
AAIAGAAVTG LLCAIVAIYL IVSSRRKKKM DGLVYHYDGN NYFVPSSQFG
GSSRNHHPPP SAIMLNSGGA .....→ continued to the kinase domain

PtEEE90055(POPTR_0008s19400g) (28 AGP motifs)

MSNSVGNPPP GSSSNESFPL QVDTNSTLSL GPSPSATDGE ESAALVDDTA
TPPPNSTNVD SPQTPEPSSP PPTSKSPPPP PPPSPPPPPP PKSNHSPPPS
PPLVSNSTKS NSSPPLKISP PPNSPPPSPN PPPTPAKKES SSSSVPSPPP
PAASPPPAGK FVPPPLSRDV QQSPPPPAEF KPSLSPPISN VSPKTLDSNS
NPSNSGRVPT DSRFHSPPVP GASPSDHPSS TSTDATNHNV PRTPPAPGNE
SNEAGGKTII AAAVGAAVTG LFLLTLIAAI FLVVKSRKKR VANASGHYMP
PKSFTLKTDG YHYGQQQQSV .....→ continued to the kinase domain

PpXP_001766257 (30 AGP motifs)

MSATPPTSGG VPPVVSPPTP NAVPPVAVNP PVVPPPVATP PTIPPATPVV
NPPTATVPPA ATPPVPVAVP PTATPPAPVA VSPALSPPAP VAVPPTLSPP
PPDALPPSVS TPPPPALSPP SPTSSSPPQP TPVPPSAPGG TPPSPILLSP
PPAVNRTSPP AALTPPASTS SNSSSTAIIA GVVGGGALLA LVALVLLFVC
CRKKRSRKDT LPYITPHGGG .....→ continued to the kinase domain

AtPERK8(AT5G38560.1) (40 AGP motifs)

MSLVPPLPIL SPPSSNSSTT APPPLQTQPT TPSAPPPVTP PPSPPQSPPP
VVSSSPPPPV VSSPPPSSSP PPSPPVITSP PPTVASSPPP PVVIASPPPS
TPATTPPAPP QTVSPPPPPD ASPSPPAPTT TNPPPKPSPS PPGETPSPPG
ETPSPPKPSP STPTPTTTTS PPPPPATSAS PPSSNPTDPS TLAPPPTPLP
VVPREKPIAK PTGPASNNGN NTLPSSSPGK SEVGTGGIVA IGVIVGLVFL
SLFVMGVWFT RKRKRKDPGT FVGYTMPPSA YSSPQGSDVV LFNSRSSAPP
KMRSHSGSDY MYASSDSGMV .....→ continued to the kinase domain

Fig. S6. The N-terminal protein structure of RHS10 homologs. Pro residues are in bold, extensin-like SPx repeats are in red, AGP repeats (AP/PA/SP/TP) are underlined, and the transmembrane domain is in green bold. The transmembrane domain was predicted by TMHMM Server v. 2.0 (http://www.cbs.dtu.dk/services/TMHMM-2.0/).

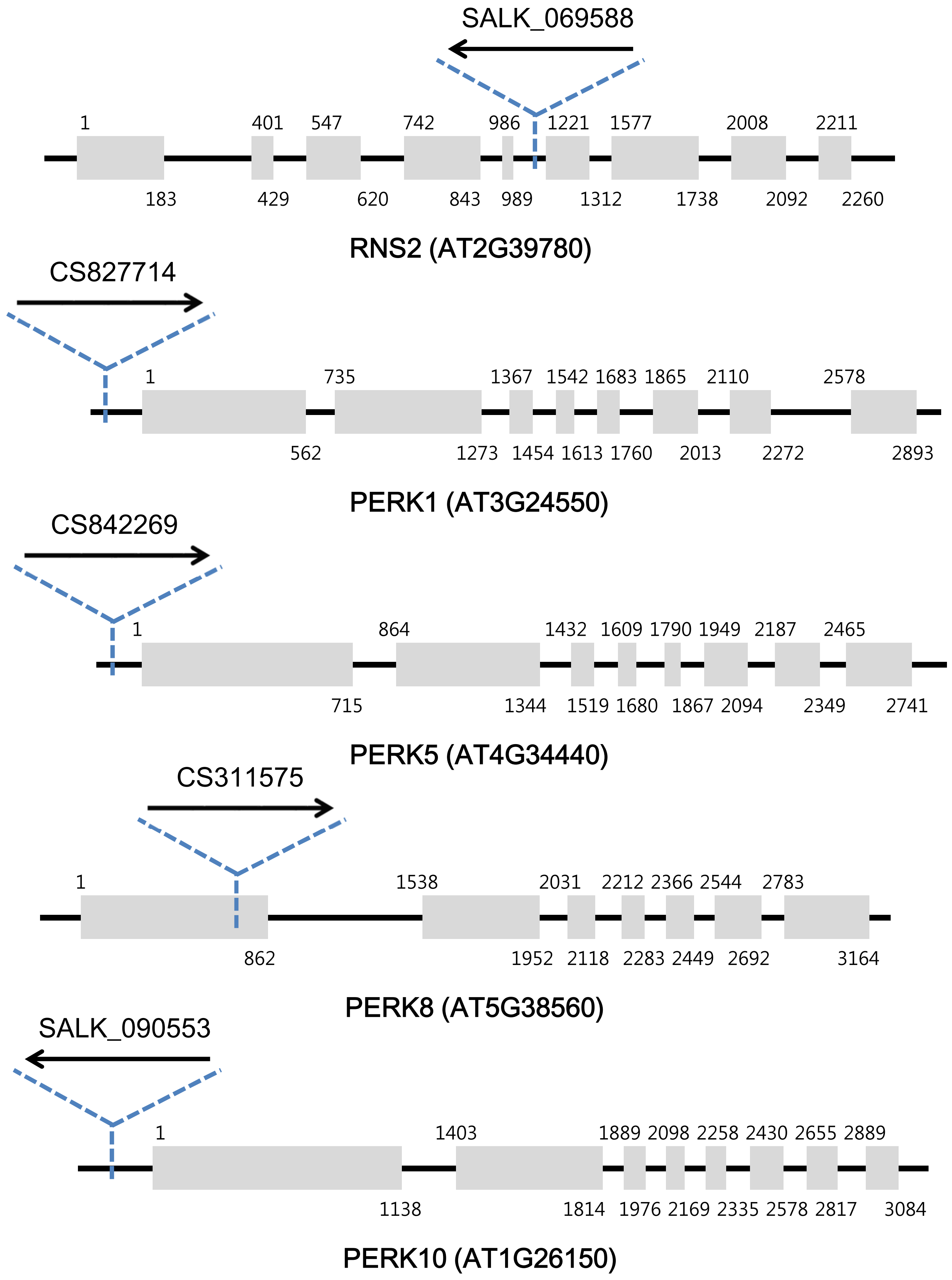

Fig. S7. T-DNA insertion positions in *rns2* and *perk* mutants. Arrows indicate the direction of T-DNA insertion (from the left border). Gray boxes represent exons, and numbers indicate the nucleotide position relatively to the start codon.

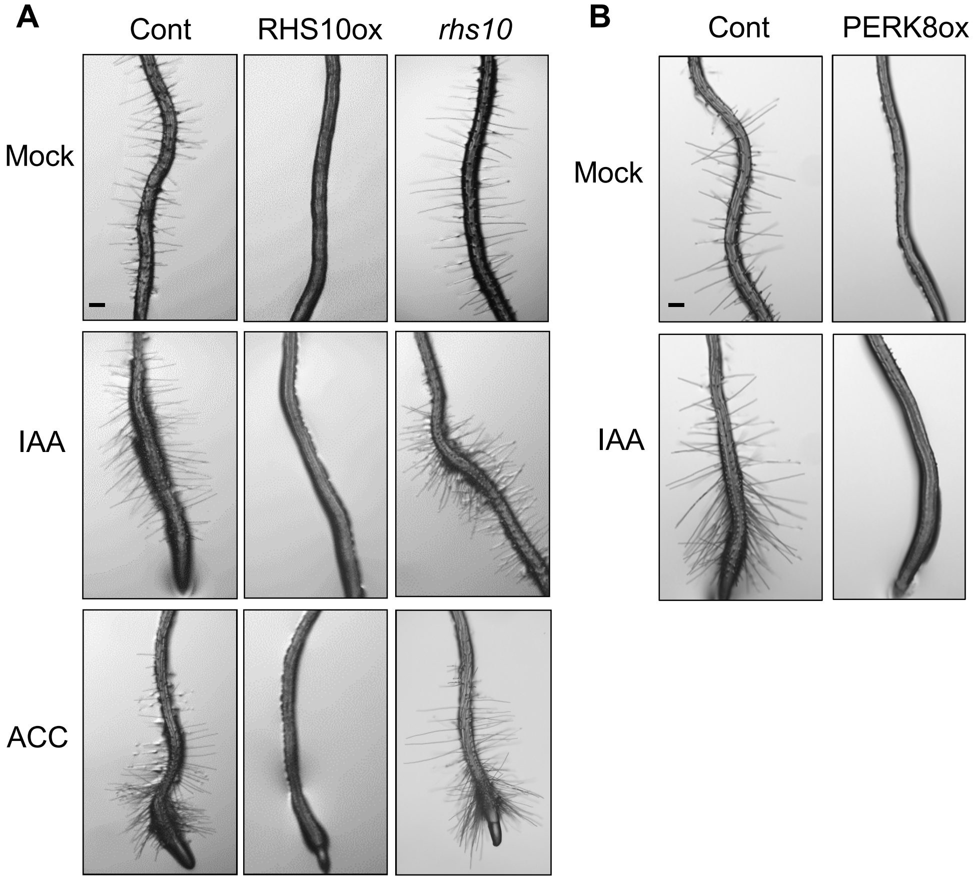

Fig. S8. Effects of auxin and an ethylene precursor on root hair restoration of RHS10ox or PERK8ox transformants. (A and B) Wild type (Cont), mutant (*rhs10*), and root hair-specific expression transformants (RHS10ox, *ProE7:RHS10*; PERK8ox, *ProE7:PERK8*) seedlings were treated without (mock) or with IAA (20 nM) or ACC (5 μM) for a day before observation. Bar is 100 μm for all.

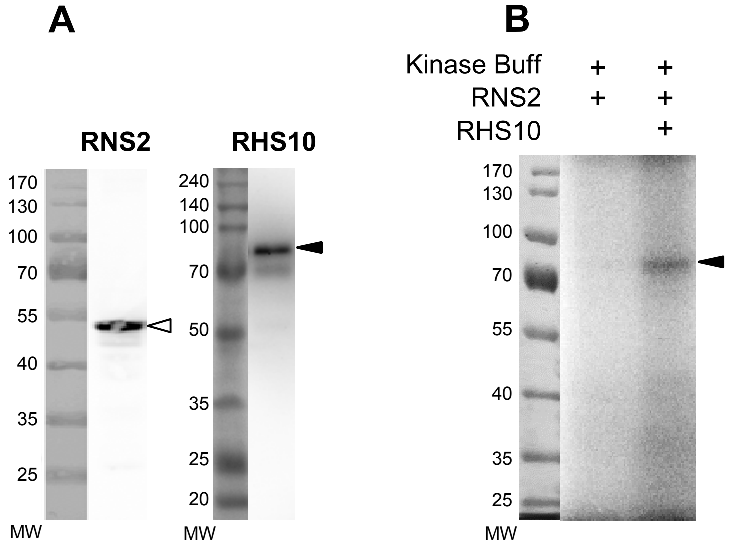

Fig. S9. Protein blot analysis and an in vitro kinase assay of RNS2 and the RHS10 kinase domain. (A) Protein blot analyses of GST-fusion proteins of RNS2 and RHS10 kinase domain using anti-GST antibody. Arrow heads indicate the fusion proteins for RNS2 (open) and RHS10 kinase domain (closed) whose calculated molecular sizes are 75.2 and 54.2 kD, respectively. (B) *In vitro* kinase assay of the RHS10 kinase domain. GST-affinity-purified RNS2 and RHS10 were used for the kinase assay including [^{32}P]γ-ATP. Auto-phosphorylated RHS10 was indicated by the arrow head, but phosphorylated RNS2 was not detectable.

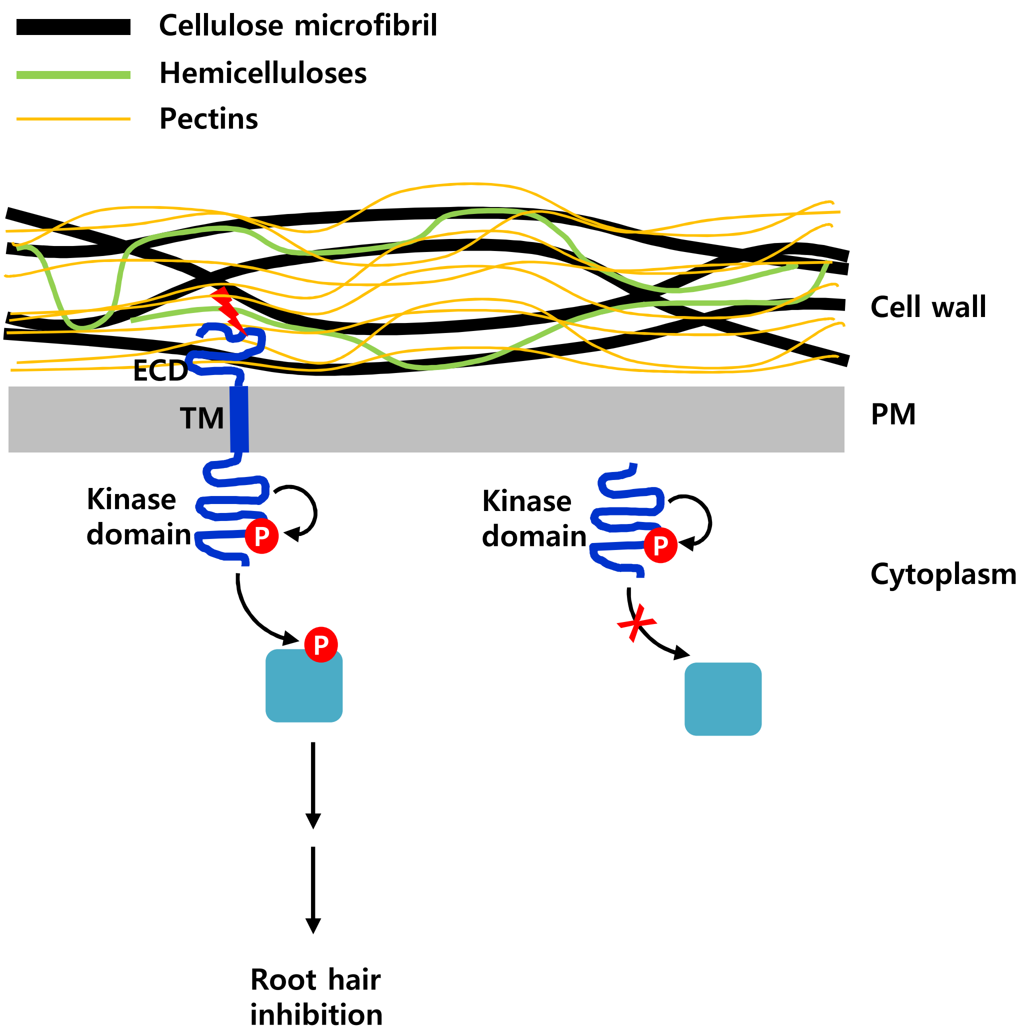

Fig. S10. A model for the role of the extracellular domain (ECD) of RHS10. RHS10 requires the ECD to phosphorylate the downstream targets and inhibit root hair growth. The kinase domain of RHS10 without the ECD only can autophosphorylate but not phosphorylate the substrate. This suggests that the cell wall signal mediated by RHS10-ECD is necessary for RHS10 to modulate root hair growth. ECD, extracellular domain; PM, plasma membrane; TM, transmembrane domain; P, phosphate

Table S1. Primer List

Subject	Primer name	Primer sequence (5′ to 3′)
RHS10-GFPox	1g70-full-SalF5	TGAAGTCGACATGGGAGATATCTGCAATGGAATC
	1g70-full-NcoR5	CTATCCATGGATCAGTAGCGCCGGTTATTGAAG
Deletion analysis of RHS10-ECD	RHS10 Sl-MF	TCGACAGGCAAAAAAATGTCGGACTCGA
	RHS10 Bg2-MR	GATCTCGAGTCCGACATTTTTTTGCCTG
	R10 Bg2-D1F	ACCAAGATCTTTTTCTCCTCCGCCAACTG
	R10 Bg2-D2F	AAAGAGATCTTCACCGCCGCCCGAGTCC
	R10 Bg2-D3F	TCCTAGATCTCCTTCAGCACCAGCCACATC
	R10 Bg2-D4F	ACCTAGATCTAGCGGTGGCGGCTATCAAG
	R10 Bg2-D5F	AAAGAGATCTATTGATGCCTACAGTGACTC
	R10 Bg2-D3-1F	AACGAGATCTCGTAACAGTTCCCACGCAC
	R10 Nc3050R	ATGCCCATGGAAGAGCTCAAGCATGAG
PERK5ox	gPERK5Sl-F	AAACGTCGACAACAACATTCAAAATGGC
	gPERK5Bm-R	TATGGGATCCGTTGTCTCTTCTTTCAAAGC
PERK8ox	gPERK8 Sl_F	AGAAGTCGACCTGCTTCAGATTCGTATTCG
	gPERK8 Bm_R	TTTAGGATCCAAGAAGAAAGAAGCAAAGGC
Os03g37120ox	Os3g37120 Bg2F	AATCAGATCTCCATTGTTGCTTCTGTGAGG
	Os3g37120 NcR	ACAACCATGTATGTGCCGTTAGTGCTCAGC
Os06g29080ox	Os06gCloningF	GATTCCTAGGCAGATCATCGAGGAGCCATTG
	Os06gCloningR	ACGTACGCGTACCGGTGCTAAGCACTTCAA
PtRHS10ox	Pt8s19400 Sl F	AGAGGTCGACGACATCCGTTAGGAAAAGGG
	Pt8s19400 KnR	AAGAGGTACCACTGTTTGGCCATGTCACCT
RNS2ox	RNS2-F-Sal	TACAGTAGACCCGATCTGAATTCGGAGCTA
	RNS2-R-Bam	CATCGGATCCGATTCAAAGAGCTTCTCTTTCTG
ROL1-Ri-1/2	rol1Ri-a1-EcoF	GTGAATTCGCTAGTGCTGACTTGGTGA
	rol1Ri-a1-XhoR	AGCCTCGAGAAGGACATGAGTTCCATAG
	rol1Ri-a2-HinF	GATAAAGCTTGTGCTGACTTGGTGAATCA
	rol1Ri-a2-XbaR	CCTCTAGAAGGACATGAGTTCCATAG
	rol1Ri-b1-XhoF	AGTCTCGAGTTAAGTATGCATACGGGCCA
	rol1Ri-b1-EcoR	TAGAATTCGAACTACAAATACACCCCT
	rol1Ri-b2-XbaF	GTCTCTAGATTAAGTATGCATACGGGCCA
	rol1Ri-b2-HinR	ATATAAGCTTCGAACTACAAATACACCCCT
LRX2-Ri	lrx2Ri-a1-XhoF	CACCTCGAGCGTCAACACCAGTTGAGTAC
	lrx2Ri-a1-EcoR	CGAATTCCTCGTATCAACCATAAGACAC
	lrx2Ri-a2-XbaF	CCATCTAGACCGTCAACACCAGTTGAGT
	lrx2Ri-a2-HinR	AAATAAGCTTAATGTCTCGTATCAACCATAAG
RT-PCR	RHS10 944sqF	ATCCTACAAAGGGATACTCTGGTCC
	RHS10_1124R	TTATGTCCGTTAGCTCTTCATATGTG
Yeast Two Hybrid	1g70-cDNA-F4-Nde	CAAACATATGTCGGACTCGCCAACTTCTTC
	1g70-cDNA-R4-Nco	CCGGCCATGGTCTTCCCTTGATAGCC
	1g70cDNA-NcoF3	GTGTCCATGGTCAGAAGAAAGAAGAAGAG
	1g70 cDNA Ps R2	TTACTGCAGTACAAAGAGCTCAAGCATGAGGG

(continued)

Table S1. The Primer List (continued)

Subject	Primer name	Primer sequence (5′ to 3′)
Kinase assay	RHS10-Kinase-Eco-FN	TATAGAATTCAAGAAGAAGAGAAACATTGATGCC
	RHS10-Kinase-Sal-R	ATATGTCGACTCAGTAGCGCCGGTTATTGA
	RHS10-321-R	ACCAAAACCTCCTTCTCCAA
	RNS2-CDS-Eco-F	TATAGAATTCATGGCGTCACGTTTATGTCTTC
	RNS2-CDS-Sal-R	ATATGTCGACCTTTTCCGATTCAAAGAGCTTCT
	RNS2-123-R	CCATTGAAGAGATAGAGCGA

Supplementary methods

Hormone treatment

For hormone response assay, seedlings grown on vertical standing 0.8% agar medium for 3 d after germination onto standing agar medium with IAA (20 nM) or ACC (5 μM) and then growing them for 1 d for the observation of phenotypic changes. For each treatment, 40 seedlings of RHS10ox were checked, with 20 seedlings of WT and *rhs10* as control.

Preparation of RHS10 and RNS2 proteins

GST-fusion proteins of RNS2 and RHS10 kinase domain were expressed in the *E. coli* (BL21DE3) cell and purified using a glutathione sepharose 4B affinity column (Elpis, Korea). Purified recombinant fusion proteins were used for protein blot analysis and *in vitro* kinase assay.

In vitro kinase assay and protein blot analysis

Purified GST-fusion proteins of RNS2 and RHS10 kinase domain were incubated in a 40 μl final volume of the kinase buffer (20 mM Pipes pH 7.0, 10 mM $MgCl_2$, 2 mM $MnCl_2$, 1 mM DTT) and 10 μCi of $[^{32}P]\gamma$ -ATP and incubated at room temperature for 30 min. The kinase reaction was stopped by adding 5 μl of 5× SDS sample buffer (1M Tris-HCl pH6.8, 20% w/v SDS, 0.05% w/v bromophenol blue), boiled for 5 min, and electrophoresed through a 10% w/v SDS-PAGE gel. The SDS-PAGE gel was stained with Coomassie blue, and the phosphorylated protein bands were detected by BAS 2500 (Fujiflim, Japan). In the protein blot analysis, the GST-fused RHS2 and RHS10 kinase domain were detected by anti-GST antibody.
